# Crystal structures of the penta- and hexa­hydrate of thulium nitrate

**DOI:** 10.1107/S2056989020015388

**Published:** 2020-11-24

**Authors:** Wilhelm Klein

**Affiliations:** a Technische Universität München, Department of Chemistry, Lichtenbergstr. 4, 85747 Garching, Germany

**Keywords:** crystal structure, thulium, nitrate, hydrate, hydrogen bonding

## Abstract

Tm(NO_3_)_3_·5H_2_O and Tm(NO_3_)_3_·6H_2_O, or more precisely [Tm(NO_3_)_3_(H_2_O)_4_]·H_2_O and [Tm(NO_3_)_3_(H_2_O)_4_]·2H_2_O, respectively, have been obtained from a concentrated solution of Tm_2_O_3_ in HNO_3_. The crystal structures of the two hydrates show strong similarities as both crystallize in space group *P*  with all atoms at general positions and contain neutral, mol­ecular [Tm(NO_3_)_3_(H_2_O)_4_] complexes, *i.e.* ten-coordinated Tm^III^ cations with three nitrate anions as bidentate ligands and four coordinating water mol­ecules, and one or two additional crystal water mol­ecules, respectively. All building units are connected by medium–strong to weak O—H⋯O hydrogen bonds. Tm(NO_3_)_3_·6H_2_O represents the maximally hydrated thulium nitrate as well as the heaviest rare earth nitrate hexa­hydrate known to date.

## Abstract 2

The title compounds represent the most hydrated thulium nitrates known so far. Both structures consists of mol­ecular [Tm(NO_3_)_3_(H_2_O)_4_] complexes and additional water mol­ecules, which are inter­connected by medium-strong to weak hydrogen bonds.

## Chemical context   

The nitrates of the rare earth metals have long been used to separate and purify these elements. For example, when thulium was discovered (Cleve, 1897 ▸), fusion of nitrates was already used to separate the element from the erbium-containing earth, and a hydrate of thulium nitrate in substance was already described more than 100 years ago with four equivalents of water of crystallization and of highly hygroscopic nature (James, 1911 ▸). Later, among others, double nitrates like Mg_3_ *Ln*_2_(NO_3_)_12_·24H_2_O and (NH_4_)_2_ *Ln*(NO_3_)_5_·4H_2_O (*Ln* = rare earth element) were used to separate the elements by means of fractional crystallization (Prandtl, 1938 ▸). Also, when more sophisticated separation procedures such as chromatographic methods and solvent extraction were developed (Bock, 1950 ▸), there was still considerable inter­est in these complex nitrates because of their high solubility even in organic solvents. Numerous structural investigations have been reported for this family, not least for the hydrated compounds (Wickleder, 2002 ▸).

Considering the structural information for the maximally hydrated rare earth nitrates, a general tendency of a decreasing amount of water with increasing atomic number is obvious: for the lighter homologues La–Nd and Sm–Tb, the hexa­hydrates are found as maximally hydrated compounds for the nitrates (La: Eriksson *et al.*, 1980 ▸; Ce: Milinski *et al.*, 1980 ▸; Pr: Decadt *et al.*, 2012 ▸; Nd: Rogers *et al.*, 1983 ▸; Sm: Kawashima *et al.*, 2000 ▸; Eu: Stumpf & Bolte, 2001 ▸; Gd: Taha *et al.*, 2012 ▸; Tb: Moret *et al.*, 1990 ▸) while for the heavier elements Dy–Er and Yb, only penta­hydrates have been reported (Ho: Rincke *et al.*, 2017 ▸; other: Junk *et al.*, 1999 ▸). Confirming this trend, the highest hydrate of Lu nitrate is the tetra­hydrate (Junk *et al.*, 1999 ▸), and for Tm the trihydrate exhibits the highest number of water mol­ecules reported so far (Riess, 2012 ▸).

In the present research communication, the new penta- and hexa­hydrates of Tm(NO_3_)_3_ are reported. While the penta­hydrate of Tm(NO_3_)_3_ fills the gap within the known compounds containing Er and Yb, the hexa­hydrate indeed represents the highest hydrated nitrate including Tm and shifts the border of known stable compounds notably to heavier rare earth elements.

## Structural commentary   

**Figure 1:**
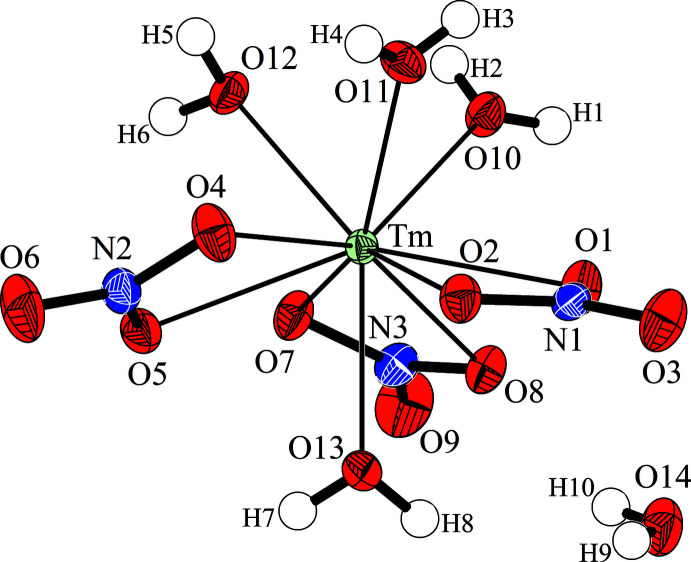
Asymmetric unit of Tm(NO_3_)_3_·5H_2_O with the atom-numbering scheme. Anisotropic displacement ellipsoids for non-H atoms are drawn at the 50% probability level.

**Figure 2:**
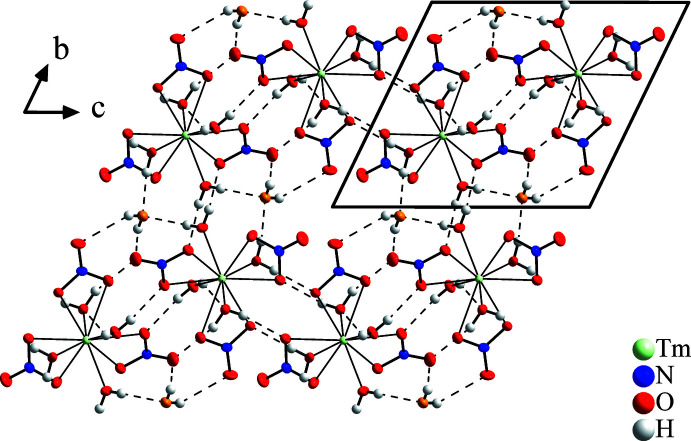
Crystal structure of Tm(NO_3_)_3_·5H_2_O in a view along [100]. Hydrogen bonds are shown as dotted lines up to an O⋯H distance of 2.45 Å. Anisotropic displacement ellipsoids of non-H atoms are drawn at the 50% probability level.

Tm(NO_3_)_3_·5H_2_O crystallizes in the Y(NO_3_)_3_·5H_2_O type of structure (Eriksson, 1982 ▸) in space group *P* 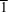 with all atoms at general positions. The structure consists of isolated mol­ecular [Tm(NO_3_)_3_(H_2_O)_4_] complexes and one additional free water mol­ecule per formula unit (Fig. 1 ▸). The nitrate anions act as bidentate ligands so the Tm^III^ atom is tenfold coordinated. The nitrate ions form an equatorial belt separating one aqua ligand from the other three, and are slightly inclined in the same sense and form a propeller-like shape. The nitrate anions coordinate asymmetrically at one shorter [2.3980 (17)–2.4479 (16) Å] and one slightly longer distance [2.5081 (16)–2.6193 (18) Å] each. The shortest Tm—O bonds [2.3235 (17)–2.3526 (18) Å] are formed with the three aqua ligands on the same side of the plane, while the remaining Tm—O(H2) bond is in the range of the shorter bonds to the nitrate ions. The anions are almost planar with an O—N—O angular sum of 360.0° where the angle formed by the coordinating O atoms is significantly reduced. The N—O bond lengths are between 1.256 (3) and 1.290 (3) Å for coordinating and 1.213 (3) and 1.220 (3) Å for non-coordinating O atoms. Within the water mol­ecules, the O—H bond lengths are between 0.68 (6) and 0.86 (4) Å, and the H—O—H angles between 102 (4) and 111 (4)°. The structural entities, *i.e*. the mol­ecular [Tm(NO_3_)_3_(H_2_O)_4_] complexes and H_2_O mol­ecules, are inter­connected by almost linear hydrogen bonds (see Fig. 2 ▸) of medium–strong to weak strength. In detail, eight of ten independent H atoms form hydrogen bonds shorter than 2.30 Å with O—H⋯O angles greater than 163°, while atoms H2 and H10 are part of bifurcated and slightly longer hydrogen bonds (Table 1 ▸).

**Figure 3:**
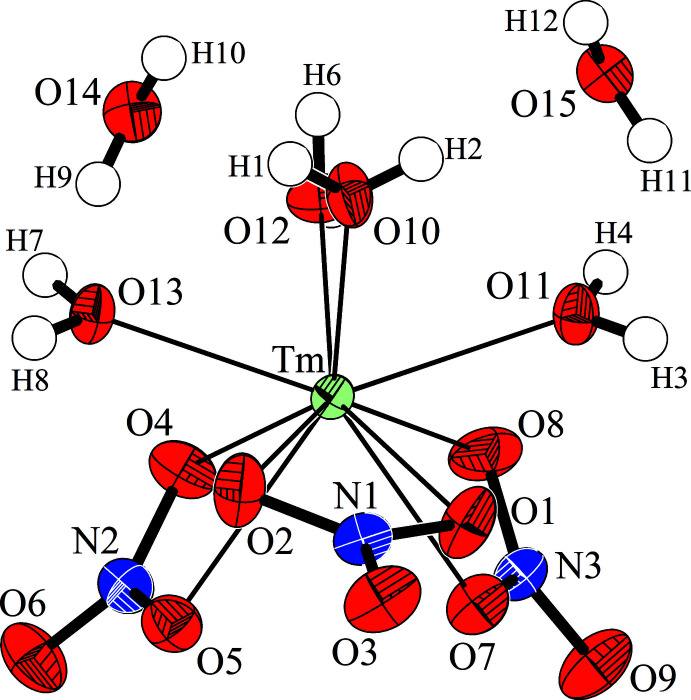
Asymmetric unit of Tm(NO_3_)_3_·6H_2_O with the atom-numbering scheme. Anisotropic displacement ellipsoids for non-H atoms are drawn at the 50% probability level.

**Figure 4:**
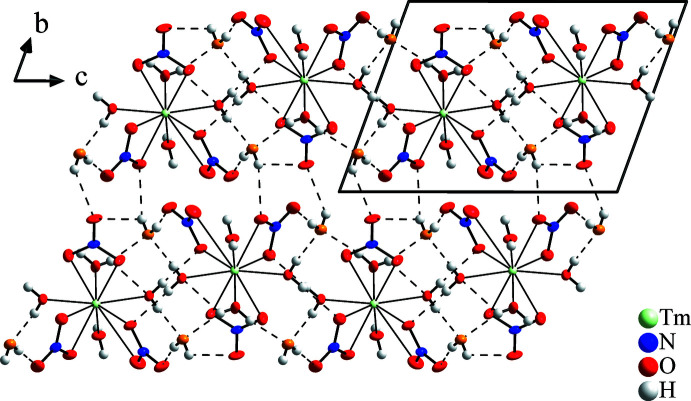
Crystal structure of Tm(NO_3_)_3_·6H_2_O in a view along [100]. Hydrogen bonds are shown as dotted lines up to an O⋯H distance of 2.5 Å. Anisotropic displacement ellipsoids of non-H atoms are drawn at the 50% probability level.

Tm(NO_3_)_3_·6H_2_O also crystallizes in space group *P* 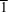 without occupying special positions and is isotypic with the respective Pr compound (Decadt *et al.*, 2012 ▸). In comparison with Tm(NO_3_)_3_·5H_2_O the volume (as determined at 223 K) increases by 34.4 Å^3^ for the two additional H_2_O mol­ecules per unit cell. Further structural similarities to the penta­hydrate include the presence of mol­ecular [Tm(NO_3_)_3_(H_2_O)_4_] complexes and free water mol­ecules, but here two per formula unit. The [Tm(NO_3_)_3_(H_2_O)_4_] complexes of the penta- and hexa­hydrates differ slightly, since in the latter the four water mol­ecules and the three nitrate ligands accumulate on opposite sides of the complex (Fig. 3 ▸). With the nitrate anions as more or less bidentate ligands, again ten atoms are found in the first coordination sphere of the Tm^III^ atom in Tm(NO_3_)_3_·6H_2_O. The resulting polyhedron can be described as a strongly distorted bicapped square anti­prism. The shortest Tm—O bonds are observed to the aqua ligands [2.2897 (18)–2.3360 (16) Å]. Similar to the penta­hydrate, the nitrate anions show an asymmetric coordination with one shorter [2.4039 (17)–2.4677 (17) Å] and one longer Tm—O distance [2.5034 (18), 2.5252 (18), 2.991 (2) Å] each. In one case, this is so severe that the corresponding Tm—O distance is even larger than the distance between the Tm^III^ atom and the central N atoms of the two remaining anions, and the arrangement should therefore rather be described as a [9 + 1] coordination. The reason for this is probably the missing space in the coordination sphere of the Tm^III^ atom, which makes such a distance increase necessary. A qualitatively analogous observation of a single extended *Ln*—N distance was made for all isotypic compounds of other rare earth elements, but the relative extension of the distance in the structure described here is much larger than in all other examples, as can be expected for the representative with the smallest ion radius so far. The consideration including a reduced coordination number for the Tm^III^ atom is supported by the different shape of the respective nitrate ion. While two anions are very similar with two longer and one shorter N—O bonds for coordinating and non-coordinating O atoms, respectively, and one reduced O—N—O angle between the coordinating O atoms, the third anion exhibits only one longer N—O bond of 1.275 (2) Å, indicating the coordinating O atom, and two shorter and almost equal N—O distances of 1.232 (3) Å and 1.236 (3) Å with more regular O—N—O angles. However, all nitrate ions are planar with an O—N—O angular sum of 360.0°. The water mol­ecules show O—H bond lengths between 0.73 (5)and 0.85 (4) Å and H—O—H angles between 105 (5) and 112 (4)°. The metal complexes and the water mol­ecules build a three-dimensional network of hydrogen bonds, again of medium–strong to weak character (Table 2 ▸, Fig. 4 ▸). Nine of twelve independent H atoms form hydrogen bonds shorter than 2.2 Å with O—H⋯O angles greater than 164° while H6, H10, and H12 are involved in weak and bifurcated hydrogen bonds.

**Figure 5:**
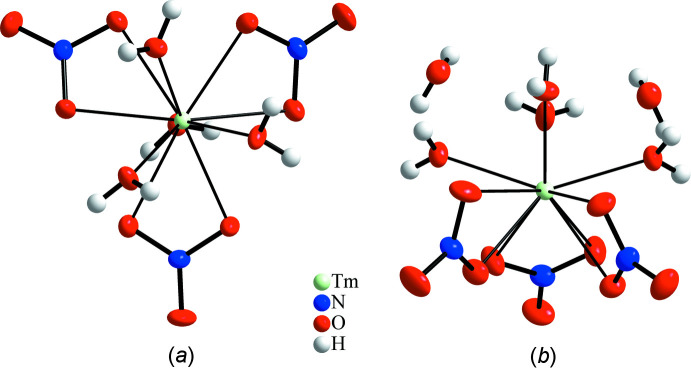
Structural details to emphasize the mol­ecular pseudo-symmetry in the title compounds: (*a*) a pseudo-threefold rotation axis in the mol­ecular complex present in Tm(NO_3_)_3_·5H_2_O; (*b*) a pseudo-mirror plane in the mol­ecular complex present in Tm(NO_3_)_3_·6H_2_O. Anisotropic displacement ellipsoids of non-H atoms are drawn at the 50% probability level.

Inter­estingly, the mol­ecular Tm complexes in both crystal structures exhibit an alleged higher symmetry, *viz*. a threefold rotation axis in the penta­hydrate and a mirror plane in the hexa­hydrate, as illustrated in Fig. 5 ▸. However, these are pseudo-symmetries, with the higher symmetry violated at a mol­ecular level and in the first coordination sphere, and incompatible with the space-group symmetry.

## Database survey   

The crystal structure of anhydrous Tm(NO_3_)_3_ was determined quite recently (Heinrichs, 2013 ▸), and one hydrated phase has been reported so far, *i.e.* the trihydrate (Riess, 2012 ▸). In addition, basic oxo-hydroxo-nitrate hydrates are known with Tm (Giester *et al.*, 2009 ▸). The thulium nitrate penta­hydrate adopts the Y(NO_3_)_3_·5H_2_O type of structure (Eriksson, 1982 ▸; Klein, 2020 ▸), and is isotypic with the respective Eu (Ribár *et al.*, 1986 ▸), Gd (Stockhause & Meyer, 1997 ▸), Dy, Er, Yb (Junk *et al.*, 1999 ▸) and Ho compounds (Rincke *et al.*, 2017 ▸). Tm(NO_3_)_3_·6H_2_O is isotypic with the nitrate hexa­hydrates of Y (Ribár *et al.*, 1980 ▸), Pr (Rumanova *et al.*, 1964 ▸; Fuller & Jacobsen, 1976 ▸; Decadt *et al.*, 2012 ▸), Nd (Rogers *et al.*, 1983 ▸; Shi & Wang, 1991 ▸), Sm (Shi & Wang, 1990 ▸; Kawashima *et al.*, 2000 ▸), Eu (Stumpf & Bolte, 2001 ▸; Ananyev *et al.*, 2016 ▸), Gd (Ma *et al.*, 1991 ▸; Taha *et al.*, 2012 ▸) and Tb (Moret *et al.*, 1990 ▸).

## Synthesis and crystallization   

[Tm(NO_3_)_3_(H_2_O)_4_]·H_2_O was prepared by dissolving Tm_2_O_3_ (Fluka AG; 99.9%) in hot aqueous nitric acid (65%_wt_). From saturated solutions, crystals with sizes up to the millimetre range were grown at room temperature within one day. Single crystals were removed, cleansed from the mother liquor and placed on a microscope slide in air. For the single-crystal data collection, crystals were immersed into perfluoro­alkyl ether, which also acts as glue on a glass tip during the measurement. The remaining crystals were carefully ground to measure an X-ray powder pattern that, according to a comparison with the pattern simulated from the single-crystal structure determination, showed exclusively reflections of the penta­hydrate. The crystals are hygroscopic and usually deliquesce within hours under ambient conditions depending on air humidity. Rapid re-crystallization within minutes can be induced by scratching on the glass slide. Surprisingly, from one recrystallization the hexa­hydrate [Tm(NO_3_)_3_(H_2_O)_4_]·2H_2_O was obtained. All investigated crystals from this batch revealed the unit cell of the hexa­hydrate, so the crystallization seemed to result in a pure product in this case as well. Optically indistinguishable, the crystals of the hexa­hydrate showed the same deliquescence behaviour. It has not been possible to determine the exact conditions required to obtain the hexa­hydrate so far. According to EDX measurements, the crystals contain Tm as the only element heavier than oxygen.

## Refinement   

Crystal data, data collection, and structure refinement details are summarized in Table 3 ▸. In both structure refinements, all hydrogen atoms have been located from difference Fourier maps and refined with free atomic coordinates and isotropic displacement parameters.

## Supplementary Material

Crystal structure: contains datablock(s) global, TONi-2_223K, TONii-3_223K. DOI: 10.1107/S2056989020015388/wm5589sup1.cif

Structure factors: contains datablock(s) TONi-2_223K. DOI: 10.1107/S2056989020015388/wm5589TONi-2_223Ksup2.hkl

Structure factors: contains datablock(s) TONii-3_223K. DOI: 10.1107/S2056989020015388/wm5589TONii-3_223Ksup3.hkl

CCDC references: 2045553, 2045552

Additional supporting information:  crystallographic information; 3D view; checkCIF report

## Figures and Tables

**Table 1:** Hydrogen-bond geometry (Å, °) for Tm(NO_3_)_3_·5H_2_O

*D*—H⋯*A*	*D*—H	H⋯*A*	*D*⋯*A*	*D*—H⋯*A*
O10—H1⋯O1^i^	0.82 (4)	2.03 (4)	2.849 (2)	174 (3)
O10—H2⋯O13^ii^	0.78 (4)	2.32 (4)	2.996 (3)	145 (3)
O10—H2⋯O2^ii^	0.78 (4)	2.48 (4)	3.035 (3)	129 (3)
O11—H3⋯O14^i^	0.77 (5)	1.98 (5)	2.739 (3)	167 (5)
O11—H4⋯O2^iii^	0.85 (4)	2.03 (4)	2.874 (2)	171 (4)
O12—H5⋯O14^iv^	0.83 (4)	1.90 (4)	2.715 (3)	165 (4)
O12—H6⋯O7^v^	0.83 (4)	1.95 (4)	2.776 (2)	174 (4)
O13—H7⋯O5^vi^	0.82 (5)	1.98 (5)	2.784 (2)	166 (4)
O13—H8⋯O3^vii^	0.86 (4)	2.12 (4)	2.953 (3)	163 (4)
O14—H9⋯O3^vii^	0.84 (4)	2.27 (5)	3.095 (3)	167 (4)
O14—H10⋯O9	0.68 (6)	2.44 (6)	3.040 (3)	148 (5)
O14—H10⋯O6^viii^	0.68 (6)	2.62 (6)	3.132 (4)	134 (5)

Symmetry codes: (i) 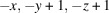; (ii) 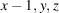; (iii) 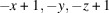; (iv) 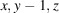; (v) 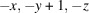; (vi) 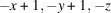; (vii) 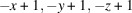; (viii) 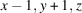.

**Table 2:** Hydrogen-bond geometry (Å, °) for Tm(NO_3_)_3_·6H_2_O

*D*—H⋯*A*	*D*—H	H⋯*A*	*D*⋯*A*	*D*—H⋯*A*
O10—H1⋯O14	0.85 (4)	1.89 (4)	2.730 (3)	170 (4)
O10—H2⋯O15	0.73 (5)	2.00 (5)	2.714 (3)	164 (5)
O11—H4⋯O15^i^	0.74 (4)	1.93 (4)	2.666 (2)	174 (4)
O11—H3⋯O7^ii^	0.81 (4)	2.20 (4)	3.006 (2)	175 (4)
O12—H5⋯O8^iii^	0.80 (4)	2.15 (4)	2.943 (3)	172 (4)
O12—H6⋯O5^iv^	0.79 (4)	2.57 (4)	3.260 (3)	147 (4)
O12—H6⋯O7^iv^	0.79 (4)	2.61 (4)	3.269 (3)	142 (4)
O13—H7⋯O14^v^	0.78 (4)	1.93 (4)	2.713 (3)	174 (4)
O13—H8⋯O5^vi^	0.83 (5)	2.13 (5)	2.963 (2)	179 (5)
O14—H9⋯O6^vi^	0.80 (4)	2.01 (4)	2.804 (3)	176 (4)
O14—H10⋯O3^iv^	0.80 (5)	2.64 (5)	3.111 (3)	119 (4)
O14—H10⋯O3^vii^	0.80 (5)	2.24 (5)	2.885 (2)	138 (4)
O15—H11⋯O9^ii^	0.79 (5)	2.01 (5)	2.782 (3)	168 (4)
O15—H12⋯O4^viii^	0.79 (5)	2.29 (5)	2.926 (2)	137 (4)
O15—H12⋯O3^iv^	0.79 (5)	2.47 (5)	2.992 (3)	125 (4)

Symmetry codes: (i) 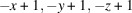; (ii) 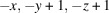; (iii) 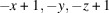; (iv) 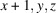; (v) 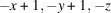; (vi) 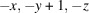; (vii) 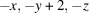; (viii) 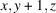.

**Table 3:** Experimental details

	Tm(NO_3_)_3_·5H_2_O	Tm(NO_3_)_3_·6H_2_O
Crystal data		
Chemical formula	[Tm(NO_3_)_3_(H_2_O)_4_]·H_2_O	[Tm(NO_3_)_3_(H_2_O)_4_]·2H_2_O
*M*_r_	445.04	463.06
Crystal system, space group	Triclinic, *P* 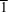	Triclinic, *P* 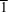
Temperature (K)	223	223
*a*, *b*, *c* (Å)	6.5782 (4), 9.5213 (5), 10.4848 (6)	6.7050 (3), 8.9733 (4), 11.4915 (6)
α, β, γ (°)	63.696 (4), 84.656 (5), 76.146 (4)	70.924 (4), 88.908 (4), 68.923 (4)
*V* (Å^3^)	571.51 (6)	605.90 (5)
*Z*	2	2
Radiation type	Mo *K*α	Mo *K*α
μ (mm^−1^)	7.85	7.41
Crystal size (mm)	0.25 × 0.2 × 0.2	0.4 × 0.2 × 0.15

Data collection		
Diffractometer	Stoe StadiVari	Stoe StadiVari
Absorption correction	Empirical (using intensity measurements) (*X-AREA*; Stoe, 2015 ▸)	Empirical (using intensity measurements) (*X-AREA*; Stoe, 2015 ▸)
*T*_min_, *T*_max_	0.798, 1.000	0.615, 1.000
No. of measured, independent and observed [*I* > 2σ(*I*)] reflections	18549, 4113, 3394	29880, 4385, 3899
*R*_int_	0.038	0.031
(sin θ/λ)_max_ (Å^−1^)	0.756	0.756

Refinement		
*R*[*F* ^2^ > 2σ(*F* ^2^)], *wR*(*F* ^2^), *S*	0.017, 0.029, 0.65	0.016, 0.035, 0.91
No. of reflections	4113	4385
No. of parameters	204	221
H-atom treatment	All H-atom parameters refined	All H-atom parameters refined
Δρ_max_, Δρ_min_ (e Å^−3^)	0.94, −0.92	1.11, −1.19

Computer programs: *X-AREA* (Stoe, 2015 ▸), *SHELXS97* (Sheldrick, 2008 ▸), *SHELXL2014/7* (Sheldrick, 2015 ▸), *DIAMOND* (Brandenburg & Putz, 2012 ▸) and *publCIF* (Westrip, 2010 ▸).

## supplementary crystallographic information

### Thulium nitrate (TONi-2_223K) Crystal data

**Table:**

[Tm(NO_3_)_3_(H_2_O)_4_]·H_2_O	*Z* = 2
*M**_r_* = 445.04	*F*(000) = 424
Triclinic, *P*1	*D*_x_ = 2.586 Mg m^−^^3^
*a* = 6.5782 (4) Å	Mo *K*α radiation, λ = 0.71073 Å
*b* = 9.5213 (5) Å	Cell parameters from 15951 reflections
*c* = 10.4848 (6) Å	θ = 3.2–36.9°
α = 63.696 (4)°	µ = 7.85 mm^−^^1^
β = 84.656 (5)°	*T* = 223 K
γ = 76.146 (4)°	Block, colourless
*V* = 571.51 (6) Å^3^	0.25 × 0.2 × 0.2 mm

### Thulium nitrate (TONi-2_223K) Data collection

**Table:**

STOE StadiVari diffractometer	4113 independent reflections
Radiation source: Genix 3D HF Mo	3394 reflections with *I* > 2σ(*I*)
Graded multilayer mirror monochromator	*R*_int_ = 0.038
Detector resolution: 5.81 pixels mm^-1^	θ_max_ = 32.5°, θ_min_ = 3.2°
ω scans	*h* = −9→9
Absorption correction: empirical (using intensity measurements) (X-AREA; Stoe, 2015)	*k* = −13→14
*T*_min_ = 0.798, *T*_max_ = 1.000	*l* = −14→15
18549 measured reflections	

### Thulium nitrate (TONi-2_223K) Refinement

**Table:**

Refinement on *F*^2^	Secondary atom site location: difference Fourier map
Least-squares matrix: full	Hydrogen site location: difference Fourier map
*R*[*F*^2^ > 2σ(*F*^2^)] = 0.017	All H-atom parameters refined
*wR*(*F*^2^) = 0.029	*w* = 1/[σ^2^(*F*_o_^2^)] where *P* = (*F*_o_^2^ + 2*F*_c_^2^)/3
*S* = 0.65	(Δ/σ)_max_ = 0.001
4113 reflections	Δρ_max_ = 0.94 e Å^−^^3^
204 parameters	Δρ_min_ = −0.92 e Å^−^^3^
0 restraints	Extinction correction: SHELXL-2014/7 (Sheldrick, 2015), Fc^*^=kFc[1+0.001xFc^2^λ^3^/sin(2θ)]^-1/4^
Primary atom site location: structure-invariant direct methods	Extinction coefficient: 0.0130 (4)

### Thulium nitrate (TONi-2_223K) Special details

**Table:**

Geometry. All esds (except the esd in the dihedral angle between two l.s. planes) are estimated using the full covariance matrix. The cell esds are taken into account individually in the estimation of esds in distances, angles and torsion angles; correlations between esds in cell parameters are only used when they are defined by crystal symmetry. An approximate (isotropic) treatment of cell esds is used for estimating esds involving l.s. planes.

### Thulium nitrate (TONi-2_223K) Fractional atomic coordinates and isotropic or equivalent isotropic displacement parameters (Å^2^)

**Table:**

	*x*	*y*	*z*	*U*_iso_*/*U*_eq_	
Tm	0.24261 (2)	0.34935 (2)	0.29463 (2)	0.01335 (4)	
N1	0.4678 (3)	0.2804 (2)	0.5557 (2)	0.0181 (4)	
O1	0.2946 (3)	0.38136 (19)	0.50762 (18)	0.0237 (3)	
O2	0.5532 (3)	0.21177 (19)	0.47971 (17)	0.0235 (3)	
O3	0.5463 (4)	0.2559 (3)	0.6667 (2)	0.0334 (5)	
N2	0.5187 (3)	0.2148 (2)	0.1245 (2)	0.0224 (4)	
O4	0.4995 (3)	0.1419 (2)	0.25849 (18)	0.0281 (4)	
O5	0.4049 (3)	0.35678 (19)	0.06621 (17)	0.0235 (3)	
O6	0.6366 (4)	0.1552 (2)	0.0567 (2)	0.0393 (5)	
N3	0.0240 (3)	0.6856 (2)	0.1466 (2)	0.0207 (4)	
O7	0.0612 (3)	0.58072 (19)	0.09609 (18)	0.0229 (4)	
O8	0.1039 (3)	0.63874 (19)	0.26683 (17)	0.0232 (3)	
O9	−0.0817 (4)	0.8199 (2)	0.0805 (2)	0.0371 (5)	
O10	−0.0906 (3)	0.3864 (2)	0.39356 (19)	0.0222 (3)	
H1	−0.150 (6)	0.447 (4)	0.428 (4)	0.034 (8)*	
H2	−0.182 (6)	0.374 (4)	0.360 (4)	0.028 (8)*	
O11	0.1965 (3)	0.1013 (2)	0.46732 (19)	0.0221 (3)	
H3	0.124 (7)	0.088 (5)	0.532 (5)	0.047 (11)*	
H4	0.278 (6)	0.014 (5)	0.475 (4)	0.035 (9)*	
O12	0.0243 (3)	0.2649 (2)	0.19433 (18)	0.0229 (3)	
H5	0.038 (6)	0.169 (5)	0.212 (4)	0.041 (10)*	
H6	0.009 (6)	0.311 (4)	0.106 (4)	0.035 (9)*	
O13	0.5125 (3)	0.5005 (2)	0.22335 (18)	0.0207 (3)	
H7	0.556 (8)	0.530 (5)	0.141 (5)	0.059 (12)*	
H8	0.495 (7)	0.586 (5)	0.237 (4)	0.045 (10)*	
O14	0.0035 (5)	0.9548 (2)	0.2793 (2)	0.0322 (5)	
H9	0.120 (7)	0.892 (5)	0.285 (4)	0.038 (9)*	
H10	−0.046 (9)	0.956 (6)	0.224 (6)	0.066 (16)*	

### Thulium nitrate (TONi-2_223K) Atomic displacement parameters (Å^2^)

**Table:**

	*U*^11^	*U*^22^	*U*^33^	*U*^12^	*U*^13^	*U*^23^
Tm	0.01449 (5)	0.01318 (5)	0.01242 (4)	−0.00290 (3)	−0.00004 (3)	−0.00565 (3)
N1	0.0201 (10)	0.0177 (8)	0.0181 (8)	−0.0059 (7)	−0.0007 (7)	−0.0082 (7)
O1	0.0221 (9)	0.0251 (8)	0.0241 (8)	0.0038 (7)	−0.0053 (6)	−0.0142 (7)
O2	0.0262 (9)	0.0215 (7)	0.0233 (8)	−0.0017 (7)	0.0005 (7)	−0.0118 (6)
O3	0.0384 (12)	0.0372 (10)	0.0277 (10)	−0.0024 (9)	−0.0141 (9)	−0.0170 (8)
N2	0.0248 (11)	0.0220 (9)	0.0214 (9)	−0.0042 (8)	0.0040 (8)	−0.0117 (8)
O4	0.0330 (10)	0.0251 (8)	0.0171 (8)	0.0029 (7)	0.0007 (7)	−0.0059 (6)
O5	0.0291 (10)	0.0197 (7)	0.0214 (8)	−0.0053 (7)	0.0058 (7)	−0.0097 (6)
O6	0.0454 (13)	0.0381 (10)	0.0335 (10)	0.0016 (9)	0.0120 (9)	−0.0224 (8)
N3	0.0231 (10)	0.0150 (8)	0.0220 (9)	−0.0010 (7)	−0.0039 (7)	−0.0072 (7)
O7	0.0329 (10)	0.0166 (7)	0.0192 (8)	−0.0016 (7)	−0.0056 (7)	−0.0084 (6)
O8	0.0266 (9)	0.0240 (8)	0.0225 (8)	−0.0042 (7)	−0.0037 (7)	−0.0131 (6)
O9	0.0451 (13)	0.0182 (8)	0.0380 (10)	0.0105 (8)	−0.0137 (9)	−0.0093 (8)
O10	0.0165 (9)	0.0288 (8)	0.0288 (9)	−0.0047 (7)	0.0026 (7)	−0.0198 (7)
O11	0.0238 (9)	0.0172 (7)	0.0205 (8)	−0.0036 (7)	0.0038 (7)	−0.0051 (6)
O12	0.0371 (10)	0.0185 (8)	0.0152 (8)	−0.0110 (7)	−0.0035 (7)	−0.0062 (6)
O13	0.0257 (9)	0.0196 (7)	0.0170 (8)	−0.0078 (6)	0.0028 (6)	−0.0073 (6)
O14	0.0520 (15)	0.0212 (9)	0.0236 (10)	−0.0045 (9)	0.0004 (9)	−0.0117 (8)

### Thulium nitrate (TONi-2_223K) Geometric parameters (Å, º)

**Table:**

Tm—O12	2.3235 (18)	N3—O9	1.216 (3)
Tm—O11	2.3235 (17)	N3—O8	1.256 (3)
Tm—O10	2.3526 (18)	N3—O7	1.290 (2)
Tm—O7	2.3980 (17)	O7—O8	2.153 (2)
Tm—O13	2.4089 (18)	O7—O9	2.184 (2)
Tm—O4	2.4181 (17)	O7—O12^vii^	2.776 (2)
Tm—O1	2.4479 (16)	O7—O12	2.778 (2)
Tm—O5	2.5081 (16)	O7—O10	3.056 (3)
Tm—O8	2.5776 (15)	O7—O13	3.150 (3)
Tm—O2	2.6193 (18)	O8—O9	2.175 (2)
Tm—N2	2.9035 (19)	O8—O10	2.738 (3)
Tm—N3	2.9212 (18)	O8—O13	2.788 (2)
Tm—N1	2.9690 (19)	O8—O3^iv^	2.969 (3)
N1—O3	1.220 (3)	O8—O14	2.982 (3)
N1—O2	1.257 (2)	O9—O14	3.040 (3)
N1—O1	1.276 (3)	O9—O6^v^	3.153 (3)
O1—O2	2.144 (2)	O9—O6^viii^	3.199 (2)
O1—O3	2.180 (3)	O10—O11	2.736 (2)
O1—O8	2.755 (2)	O10—O12	2.775 (2)
O1—O10^i^	2.849 (2)	O10—O1^i^	2.849 (2)
O1—O10	2.882 (3)	O10—O13^ix^	2.996 (3)
O1—O13	3.035 (2)	O10—O2^ix^	3.035 (3)
O1—O11	3.095 (2)	O10—H1	0.82 (4)
O2—O3	2.174 (2)	O10—H2	0.78 (4)
O2—O4	2.753 (2)	O11—O14^i^	2.739 (3)
O2—O11	2.827 (3)	O11—O12	2.777 (2)
O2—O13	2.844 (2)	O11—O2^ii^	2.874 (2)
O2—O11^ii^	2.874 (2)	O11—O4^ii^	3.253 (2)
O2—O10^iii^	3.035 (3)	O11—H3	0.77 (5)
O3—O13^iv^	2.953 (3)	O11—H4	0.85 (4)
O3—O8^iv^	2.969 (3)	O12—O14^x^	2.715 (3)
O3—O14^iv^	3.095 (3)	O12—O7^vii^	2.776 (2)
N2—O6	1.213 (3)	O12—O9^vii^	3.288 (3)
N2—O4	1.271 (3)	O12—H5	0.83 (4)
N2—O5	1.277 (3)	O12—H6	0.83 (4)
O4—O5	2.146 (2)	O13—O5^v^	2.784 (2)
O4—O6	2.184 (2)	O13—O3^iv^	2.953 (3)
O4—O11	2.779 (3)	O13—O10^iii^	2.996 (3)
O4—O12	3.088 (3)	O13—O6^v^	3.286 (3)
O5—O6	2.181 (2)	O13—H7	0.82 (5)
O5—O13^v^	2.784 (2)	O13—H8	0.86 (4)
O5—O13	2.786 (2)	O14—O12^xi^	2.715 (3)
O5—O7	2.812 (2)	O14—O11^i^	2.739 (3)
O5—O12	2.867 (3)	O14—O3^iv^	3.095 (3)
O5—O5^v^	2.981 (3)	O14—O6^viii^	3.132 (4)
O6—O14^vi^	3.132 (4)	O14—H9	0.84 (4)
O6—O9^v^	3.153 (3)	O14—H10	0.68 (6)
O6—O9^vi^	3.199 (2)		
			
O12—Tm—O11	73.38 (6)	N3—O8—O1	146.83 (13)
O12—Tm—O10	72.80 (7)	O7—O8—O1	114.60 (8)
O11—Tm—O10	71.63 (6)	O9—O8—O1	171.15 (11)
O12—Tm—O7	72.07 (6)	Tm—O8—O1	54.53 (5)
O11—Tm—O7	140.36 (7)	O10—O8—O1	63.30 (7)
O10—Tm—O7	80.06 (7)	N3—O8—O13	100.19 (13)
O12—Tm—O13	139.17 (6)	O7—O8—O13	78.06 (8)
O11—Tm—O13	137.72 (6)	O9—O8—O13	117.62 (10)
O10—Tm—O13	133.41 (6)	Tm—O8—O13	53.17 (5)
O7—Tm—O13	81.90 (6)	O10—O8—O13	104.61 (7)
O12—Tm—O4	81.26 (7)	O1—O8—O13	66.39 (6)
O11—Tm—O4	71.73 (6)	N3—O8—O3^iv^	127.07 (15)
O10—Tm—O4	139.74 (7)	O7—O8—O3^iv^	130.96 (11)
O7—Tm—O4	120.66 (6)	O9—O8—O3^iv^	113.90 (10)
O13—Tm—O4	85.91 (6)	Tm—O8—O3^iv^	107.97 (7)
O12—Tm—O1	142.71 (7)	O10—O8—O3^iv^	137.66 (9)
O11—Tm—O1	80.83 (6)	O1—O8—O3^iv^	74.92 (7)
O10—Tm—O1	73.77 (7)	O13—O8—O3^iv^	61.63 (7)
O7—Tm—O1	117.47 (5)	N3—O8—O14	97.81 (13)
O13—Tm—O1	77.34 (6)	O7—O8—O14	130.15 (10)
O4—Tm—O1	115.79 (6)	O9—O8—O14	70.27 (8)
O12—Tm—O5	72.69 (6)	Tm—O8—O14	168.88 (9)
O11—Tm—O5	116.78 (6)	O10—O8—O14	129.72 (9)
O10—Tm—O5	139.64 (7)	O1—O8—O14	115.20 (8)
O7—Tm—O5	69.91 (6)	O13—O8—O14	120.95 (9)
O13—Tm—O5	69.01 (6)	O3^iv^—O8—O14	62.67 (8)
O4—Tm—O5	51.62 (5)	N3—O9—O8	28.84 (12)
O1—Tm—O5	144.29 (6)	N3—O9—O7	30.33 (11)
O12—Tm—O8	113.85 (6)	O8—O9—O7	59.18 (7)
O11—Tm—O8	132.83 (6)	N3—O9—O14	95.93 (15)
O10—Tm—O8	67.31 (6)	O8—O9—O14	67.39 (8)
O7—Tm—O8	51.11 (5)	O7—O9—O14	125.90 (11)
O13—Tm—O8	67.90 (6)	N3—O9—O6^v^	76.99 (16)
O4—Tm—O8	152.92 (6)	O8—O9—O6^v^	82.55 (9)
O1—Tm—O8	66.42 (5)	O7—O9—O6^v^	74.67 (9)
O5—Tm—O8	109.44 (5)	O14—O9—O6^v^	91.35 (9)
O12—Tm—O2	136.38 (6)	N3—O9—O6^viii^	152.81 (17)
O11—Tm—O2	69.47 (6)	O8—O9—O6^viii^	124.77 (10)
O10—Tm—O2	114.60 (6)	O7—O9—O6^viii^	170.46 (13)
O7—Tm—O2	149.67 (6)	O14—O9—O6^viii^	60.20 (7)
O13—Tm—O2	68.73 (6)	O6^v^—O9—O6^viii^	113.72 (8)
O4—Tm—O2	66.13 (6)	Tm—O10—O11	53.69 (5)
O1—Tm—O2	49.93 (5)	Tm—O10—O8	60.27 (6)
O5—Tm—O2	104.73 (6)	O11—O10—O8	110.30 (8)
O8—Tm—O2	108.01 (5)	Tm—O10—O12	53.11 (5)
O12—Tm—N2	75.56 (6)	O11—O10—O12	60.49 (6)
O11—Tm—N2	94.20 (6)	O8—O10—O12	96.36 (7)
O10—Tm—N2	147.88 (7)	Tm—O10—O1^i^	130.59 (9)
O7—Tm—N2	95.49 (6)	O11—O10—O1^i^	143.50 (9)
O13—Tm—N2	76.27 (6)	O8—O10—O1^i^	73.51 (7)
O4—Tm—N2	25.62 (6)	O12—O10—O1^i^	155.76 (9)
O1—Tm—N2	133.73 (6)	Tm—O10—O1	54.63 (5)
O5—Tm—N2	26.00 (6)	O11—O10—O1	66.79 (7)
O8—Tm—N2	132.96 (6)	O8—O10—O1	58.62 (6)
O2—Tm—N2	85.07 (6)	O12—O10—O1	106.09 (8)
O12—Tm—N3	92.45 (6)	O1^i^—O10—O1	87.67 (7)
O11—Tm—N3	142.15 (6)	Tm—O10—O13^ix^	122.60 (8)
O10—Tm—N3	70.66 (6)	O11—O10—O13^ix^	128.05 (9)
O7—Tm—N3	25.75 (6)	O8—O10—O13^ix^	103.81 (7)
O13—Tm—N3	74.57 (6)	O12—O10—O13^ix^	78.12 (7)
O4—Tm—N3	141.95 (6)	O1^i^—O10—O13^ix^	82.96 (7)
O1—Tm—N3	91.72 (5)	O1—O10—O13^ix^	162.00 (8)
O5—Tm—N3	90.60 (5)	Tm—O10—O2^ix^	135.89 (8)
O8—Tm—N3	25.43 (5)	O11—O10—O2^ix^	90.74 (7)
O2—Tm—N3	131.08 (5)	O8—O10—O2^ix^	158.09 (8)
N2—Tm—N3	116.50 (6)	O12—O10—O2^ix^	88.57 (7)
O12—Tm—N1	145.99 (6)	O1^i^—O10—O2^ix^	93.44 (7)
O11—Tm—N1	72.73 (6)	O1—O10—O2^ix^	140.06 (8)
O10—Tm—N1	93.76 (6)	O13^ix^—O10—O2^ix^	56.26 (6)
O7—Tm—N1	137.60 (5)	Tm—O10—O7	50.62 (5)
O13—Tm—N1	72.24 (6)	O11—O10—O7	100.01 (7)
O4—Tm—N1	90.89 (6)	O8—O10—O7	43.18 (5)
O1—Tm—N1	24.96 (5)	O12—O10—O7	56.67 (6)
O5—Tm—N1	126.60 (6)	O1^i^—O10—O7	105.10 (7)
O8—Tm—N1	87.63 (5)	O1—O10—O7	88.42 (7)
O2—Tm—N1	25.01 (5)	O13^ix^—O10—O7	79.30 (7)
N2—Tm—N1	109.73 (6)	O2^ix^—O10—O7	129.22 (8)
N3—Tm—N1	112.83 (5)	Tm—O10—H1	133 (3)
O3—N1—O2	122.7 (2)	O11—O10—H1	140 (2)
O3—N1—O1	121.65 (19)	O8—O10—H1	77 (3)
O2—N1—O1	115.65 (18)	O12—O10—H1	159 (2)
O3—N1—Tm	175.24 (17)	O1^i^—O10—H1	5 (2)
O2—N1—Tm	61.74 (11)	O1—O10—H1	88 (3)
O1—N1—Tm	54.03 (10)	O13^ix^—O10—H1	84 (3)
N1—O1—O2	31.90 (10)	O2^ix^—O10—H1	91 (3)
N1—O1—O3	28.46 (11)	O7—O10—H1	110 (2)
O2—O1—O3	60.35 (8)	Tm—O10—H2	117 (2)
N1—O1—Tm	101.01 (12)	O11—O10—H2	103 (2)
O2—O1—Tm	69.19 (6)	O8—O10—H2	125 (3)
O3—O1—Tm	129.44 (9)	O12—O10—H2	64 (2)
N1—O1—O8	142.91 (14)	O1^i^—O10—H2	103 (2)
O2—O1—O8	117.71 (9)	O1—O10—H2	169 (2)
O3—O1—O8	152.42 (12)	O13^ix^—O10—H2	26 (2)
Tm—O1—O8	59.05 (5)	O2^ix^—O10—H2	39 (3)
N1—O1—O10^i^	125.68 (13)	O7—O10—H2	90 (3)
O2—O1—O10^i^	155.65 (10)	H1—O10—H2	103 (4)
O3—O1—O10^i^	97.77 (9)	Tm—O11—O10	54.68 (5)
Tm—O1—O10^i^	132.31 (7)	Tm—O11—O14^i^	126.09 (8)
O8—O1—O10^i^	76.13 (6)	O10—O11—O14^i^	79.46 (8)
N1—O1—O10	136.42 (14)	Tm—O11—O12	53.31 (5)
O2—O1—O10	111.95 (9)	O10—O11—O12	60.44 (6)
O3—O1—O10	149.48 (11)	O14^i^—O11—O12	128.28 (10)
Tm—O1—O10	51.60 (5)	Tm—O11—O4	55.71 (5)
O8—O1—O10	58.08 (6)	O10—O11—O4	108.60 (8)
O10^i^—O1—O10	92.33 (7)	O14^i^—O11—O4	163.22 (11)
N1—O1—O13	85.66 (12)	O12—O11—O4	67.54 (7)
O2—O1—O13	63.91 (7)	Tm—O11—O2	60.20 (5)
O3—O1—O13	105.20 (10)	O10—O11—O2	97.60 (7)
Tm—O1—O13	50.75 (5)	O14^i^—O11—O2	106.22 (9)
O8—O1—O13	57.34 (6)	O12—O11—O2	110.01 (8)
O10^i^—O1—O13	117.55 (8)	O4—O11—O2	58.83 (7)
O10—O1—O13	95.28 (7)	Tm—O11—O2^ii^	128.10 (9)
N1—O1—O11	82.07 (12)	O10—O11—O2^ii^	169.77 (9)
O2—O1—O11	62.22 (7)	O14^i^—O11—O2^ii^	102.25 (8)
O3—O1—O11	101.73 (9)	O12—O11—O2^ii^	112.15 (8)
Tm—O1—O11	47.83 (4)	O4—O11—O2^ii^	72.52 (7)
O8—O1—O11	100.20 (7)	O2—O11—O2^ii^	91.62 (7)
O10^i^—O1—O11	138.47 (9)	Tm—O11—O1	51.34 (5)
O10—O1—O11	54.35 (6)	O10—O11—O1	58.86 (6)
O13—O1—O11	92.12 (6)	O14^i^—O11—O1	82.73 (7)
N1—O2—O1	32.45 (11)	O12—O11—O1	100.57 (7)
N1—O2—O3	28.19 (11)	O4—O11—O1	88.96 (7)
O1—O2—O3	60.63 (8)	O2—O11—O1	42.16 (5)
N1—O2—Tm	93.26 (12)	O2^ii^—O11—O1	131.25 (8)
O1—O2—Tm	60.88 (6)	Tm—O11—O4^ii^	126.48 (8)
O3—O2—Tm	121.43 (9)	O10—O11—O4^ii^	135.39 (8)
N1—O2—O4	145.77 (15)	O14^i^—O11—O4^ii^	67.19 (8)
O1—O2—O4	114.04 (9)	O12—O11—O4^ii^	163.11 (9)
O3—O2—O4	170.50 (12)	O4—O11—O4^ii^	98.00 (7)
Tm—O2—O4	53.43 (5)	O2—O11—O4^ii^	66.31 (6)
N1—O2—O11	94.41 (14)	O2^ii^—O11—O4^ii^	52.98 (5)
O1—O2—O11	75.62 (8)	O1—O11—O4^ii^	87.35 (6)
O3—O2—O11	110.77 (10)	Tm—O11—H3	125 (3)
Tm—O2—O11	50.33 (5)	O10—O11—H3	75 (3)
O4—O2—O11	59.72 (7)	O14^i^—O11—H3	9 (3)
N1—O2—O13	94.71 (12)	O12—O11—H3	120 (3)
O1—O2—O13	73.45 (7)	O4—O11—H3	172 (3)
O3—O2—O13	111.95 (9)	O2—O11—H3	114 (3)
Tm—O2—O13	52.13 (5)	O2^ii^—O11—H3	105 (3)
O4—O2—O13	71.96 (6)	O1—O11—H3	87 (3)
O11—O2—O13	102.24 (7)	O4^ii^—O11—H3	75 (3)
N1—O2—O11^ii^	135.37 (13)	Tm—O11—H4	122 (3)
O1—O2—O11^ii^	156.10 (10)	O10—O11—H4	169 (2)
O3—O2—O11^ii^	110.89 (9)	O14^i^—O11—H4	108 (2)
Tm—O2—O11^ii^	121.11 (7)	O12—O11—H4	109 (2)
O4—O2—O11^ii^	70.59 (6)	O4—O11—H4	66 (2)
O11—O2—O11^ii^	88.37 (7)	O2—O11—H4	88 (3)
O13—O2—O11^ii^	128.19 (8)	O2^ii^—O11—H4	6 (3)
N1—O2—O10^iii^	93.92 (13)	O1—O11—H4	129 (3)
O1—O2—O10^iii^	103.88 (9)	O4^ii^—O11—H4	56 (2)
O3—O2—O10^iii^	83.51 (9)	H3—O11—H4	111 (4)
Tm—O2—O10^iii^	113.27 (7)	Tm—O12—O14^x^	125.11 (10)
O4—O2—O10^iii^	105.78 (8)	Tm—O12—O10	54.08 (5)
O11—O2—O10^iii^	162.02 (8)	O14^x^—O12—O10	116.16 (9)
O13—O2—O10^iii^	61.18 (6)	Tm—O12—O7^vii^	120.70 (8)
O11^ii^—O2—O10^iii^	96.80 (7)	O14^x^—O12—O7^vii^	105.94 (8)
N1—O3—O2	29.13 (11)	O10—O12—O7^vii^	128.10 (8)
N1—O3—O1	29.90 (11)	Tm—O12—O11	53.31 (5)
O2—O3—O1	59.02 (8)	O14^x^—O12—O11	75.07 (8)
N1—O3—O13^iv^	123.70 (15)	O10—O12—O11	59.06 (6)
O2—O3—O13^iv^	146.33 (11)	O7^vii^—O12—O11	167.99 (10)
O1—O3—O13^iv^	96.81 (9)	Tm—O12—O7	55.21 (5)
N1—O3—O8^iv^	133.41 (18)	O14^x^—O12—O7	176.85 (10)
O2—O3—O8^iv^	120.98 (12)	O10—O12—O7	66.77 (6)
O1—O3—O8^iv^	132.24 (11)	O7^vii^—O12—O7	72.20 (7)
O13^iv^—O3—O8^iv^	56.18 (6)	O11—O12—O7	106.19 (8)
N1—O3—O14^iv^	113.40 (16)	Tm—O12—O5	56.63 (5)
O2—O3—O14^iv^	84.49 (9)	O14^x^—O12—O5	117.49 (9)
O1—O3—O14^iv^	142.73 (10)	O10—O12—O5	107.92 (8)
O13^iv^—O3—O14^iv^	112.24 (8)	O7^vii^—O12—O5	75.17 (7)
O8^iv^—O3—O14^iv^	58.87 (6)	O11—O12—O5	93.64 (8)
O6—N2—O4	122.98 (19)	O7—O12—O5	59.73 (6)
O6—N2—O5	122.2 (2)	Tm—O12—O4	50.70 (5)
O4—N2—O5	114.76 (17)	O14^x^—O12—O4	87.49 (9)
O6—N2—Tm	178.28 (17)	O10—O12—O4	99.49 (7)
O4—N2—Tm	55.33 (10)	O7^vii^—O12—O4	111.72 (8)
O5—N2—Tm	59.44 (10)	O11—O12—O4	56.27 (6)
N2—O4—O5	32.70 (10)	O7—O12—O4	90.85 (7)
N2—O4—O6	27.78 (11)	O5—O12—O4	42.04 (5)
O5—O4—O6	60.48 (8)	Tm—O12—O9^vii^	131.81 (9)
N2—O4—Tm	99.05 (12)	O14^x^—O12—O9^vii^	70.05 (7)
O5—O4—Tm	66.35 (6)	O10—O12—O9^vii^	168.34 (9)
O6—O4—Tm	126.84 (9)	O7^vii^—O12—O9^vii^	41.15 (5)
N2—O4—O2	133.14 (15)	O11—O12—O9^vii^	132.43 (8)
O5—O4—O2	111.36 (9)	O7—O12—O9^vii^	107.30 (7)
O6—O4—O2	141.71 (12)	O5—O12—O9^vii^	75.57 (7)
Tm—O4—O2	60.45 (5)	O4—O12—O9^vii^	90.46 (7)
N2—O4—O11	140.81 (15)	Tm—O12—H5	123 (3)
O5—O4—O11	112.74 (9)	O14^x^—O12—H5	10 (3)
O6—O4—O11	156.42 (13)	O10—O12—H5	124 (3)
Tm—O4—O11	52.55 (5)	O7^vii^—O12—H5	102 (3)
O2—O4—O11	61.45 (6)	O11—O12—H5	77 (3)
N2—O4—O12	84.97 (14)	O7—O12—H5	166 (3)
O5—O4—O12	63.46 (7)	O5—O12—H5	107 (3)
O6—O4—O12	104.30 (10)	O4—O12—H5	80 (3)
Tm—O4—O12	48.04 (5)	O9^vii^—O12—H5	63 (3)
O2—O4—O12	103.44 (7)	Tm—O12—H6	118 (3)
O11—O4—O12	56.19 (6)	O14^x^—O12—H6	107 (2)
N2—O5—O4	32.54 (11)	O10—O12—H6	130 (2)
N2—O5—O6	28.07 (11)	O7^vii^—O12—H6	4 (3)
O4—O5—O6	60.61 (8)	O11—O12—H6	164 (3)
N2—O5—Tm	94.56 (12)	O7—O12—H6	71 (2)
O4—O5—Tm	62.03 (6)	O5—O12—H6	71 (3)
O6—O5—Tm	122.63 (9)	O4—O12—H6	107 (3)
N2—O5—O13^v^	109.94 (13)	O9^vii^—O12—H6	40 (2)
O4—O5—O13^v^	142.41 (9)	H5—O12—H6	102 (4)
O6—O5—O13^v^	81.90 (8)	Tm—O13—O5^v^	116.85 (8)
Tm—O5—O13^v^	155.31 (8)	Tm—O13—O5	57.18 (5)
N2—O5—O13	102.32 (14)	O5^v^—O13—O5	64.70 (7)
O4—O5—O13	82.54 (8)	Tm—O13—O8	58.93 (5)
O6—O5—O13	117.15 (11)	O5^v^—O13—O8	108.57 (8)
Tm—O5—O13	53.82 (5)	O5—O13—O8	96.27 (7)
O13^v^—O5—O13	115.30 (7)	Tm—O13—O2	59.14 (5)
N2—O5—O7	146.29 (14)	O5^v^—O13—O2	147.07 (8)
O4—O5—O7	114.45 (9)	O5—O13—O2	92.32 (7)
O6—O5—O7	170.40 (11)	O8—O13—O2	96.59 (7)
Tm—O5—O7	53.21 (5)	Tm—O13—O3^iv^	113.42 (8)
O13^v^—O5—O7	103.03 (7)	O5^v^—O13—O3^iv^	108.73 (8)
O13—O5—O7	68.48 (6)	O5—O13—O3^iv^	155.31 (9)
N2—O5—O12	94.81 (14)	O8—O13—O3^iv^	62.20 (7)
O4—O5—O12	74.50 (8)	O2—O13—O3^iv^	101.59 (7)
O6—O5—O12	111.87 (10)	Tm—O13—O10^iii^	121.66 (7)
Tm—O5—O12	50.68 (5)	O5^v^—O13—O10^iii^	110.19 (8)
O13^v^—O5—O12	126.93 (9)	O5—O13—O10^iii^	124.94 (8)
O13—O5—O12	103.34 (7)	O8—O13—O10^iii^	132.34 (8)
O7—O5—O12	58.56 (6)	O2—O13—O10^iii^	62.57 (6)
N2—O5—O5^v^	121.20 (17)	O3^iv^—O13—O10^iii^	79.74 (7)
O4—O5—O5^v^	128.22 (13)	Tm—O13—O1	51.90 (5)
O6—O5—O5^v^	107.16 (11)	O5^v^—O13—O1	163.64 (9)
Tm—O5—O5^v^	107.25 (8)	O5—O13—O1	108.19 (7)
O13^v^—O5—O5^v^	57.69 (6)	O8—O13—O1	56.27 (6)
O13—O5—O5^v^	57.60 (7)	O2—O13—O1	42.63 (5)
O7—O5—O5^v^	82.40 (7)	O3^iv^—O13—O1	71.17 (7)
O12—O5—O5^v^	140.96 (9)	O10^iii^—O13—O1	86.04 (6)
N2—O6—O5	29.68 (12)	Tm—O13—O7	48.90 (5)
N2—O6—O4	29.23 (11)	O5^v^—O13—O7	79.82 (7)
O5—O6—O4	58.91 (7)	O5—O13—O7	56.15 (6)
N2—O6—O14^vi^	99.61 (16)	O8—O13—O7	41.95 (5)
O5—O6—O14^vi^	122.89 (10)	O2—O13—O7	107.68 (8)
O4—O6—O14^vi^	75.58 (9)	O3^iv^—O13—O7	99.84 (7)
N2—O6—O9^v^	136.91 (18)	O10^iii^—O13—O7	169.66 (8)
O5—O6—O9^v^	124.28 (11)	O1—O13—O7	84.06 (7)
O4—O6—O9^v^	134.81 (12)	Tm—O13—O6^v^	111.12 (7)
O14^vi^—O6—O9^v^	67.17 (7)	O5^v^—O13—O6^v^	41.08 (5)
N2—O6—O9^vi^	142.10 (16)	O5—O13—O6^v^	86.61 (7)
O5—O6—O9^vi^	169.33 (12)	O8—O13—O6^v^	71.96 (6)
O4—O6—O9^vi^	113.34 (10)	O2—O13—O6^v^	168.28 (9)
O14^vi^—O6—O9^vi^	57.38 (6)	O3^iv^—O13—O6^v^	75.62 (6)
O9^v^—O6—O9^vi^	66.27 (8)	O10^iii^—O13—O6^v^	127.02 (8)
O9—N3—O8	123.31 (19)	O1—O13—O6^v^	126.97 (7)
O9—N3—O7	121.3 (2)	O7—O13—O6^v^	62.22 (6)
O8—N3—O7	115.44 (17)	Tm—O13—H7	119 (3)
O9—N3—Tm	173.32 (18)	O5^v^—O13—H7	10 (3)
O8—N3—Tm	61.80 (10)	O5—O13—H7	64 (3)
O7—N3—Tm	53.84 (10)	O8—O13—H7	119 (3)
N3—O7—O8	31.79 (10)	O2—O13—H7	138 (3)
N3—O7—O9	28.42 (11)	O3^iv^—O13—H7	114 (3)
O8—O7—O9	60.21 (8)	O10^iii^—O13—H7	102 (3)
N3—O7—Tm	100.41 (12)	O1—O13—H7	171 (3)
O8—O7—Tm	68.76 (6)	O7—O13—H7	88 (3)
O9—O7—Tm	128.67 (10)	O6^v^—O13—H7	51 (3)
N3—O7—O12^vii^	109.38 (13)	Tm—O13—H8	119 (3)
O8—O7—O12^vii^	139.23 (9)	O5^v^—O13—H8	97 (3)
O9—O7—O12^vii^	82.11 (8)	O5—O13—H8	148 (3)
Tm—O7—O12^vii^	148.08 (8)	O8—O13—H8	63 (3)
N3—O7—O12	135.69 (15)	O2—O13—H8	114 (3)
O8—O7—O12	112.27 (9)	O3^iv^—O13—H8	12 (3)
O9—O7—O12	146.72 (12)	O10^iii^—O13—H8	86 (3)
Tm—O7—O12	52.72 (5)	O1—O13—H8	82 (3)
O12^vii^—O7—O12	107.80 (7)	O7—O13—H8	96 (3)
N3—O7—O5	138.14 (16)	O6^v^—O13—H8	64 (3)
O8—O7—O5	112.81 (9)	H7—O13—H8	103 (4)
O9—O7—O5	151.26 (12)	O12^xi^—O14—O11^i^	99.62 (8)
Tm—O7—O5	56.88 (5)	O12^xi^—O14—O8	155.24 (10)
O12^vii^—O7—O5	92.35 (7)	O11^i^—O14—O8	104.73 (8)
O12—O7—O5	61.71 (6)	O12^xi^—O14—O9	124.30 (10)
N3—O7—O10	79.13 (13)	O11^i^—O14—O9	123.04 (11)
O8—O7—O10	60.53 (7)	O8—O14—O9	42.34 (5)
O9—O7—O10	97.66 (9)	O12^xi^—O14—O3^iv^	108.74 (10)
Tm—O7—O10	49.32 (5)	O11^i^—O14—O3^iv^	109.99 (9)
O12^vii^—O7—O10	146.96 (10)	O8—O14—O3^iv^	58.46 (7)
O12—O7—O10	56.57 (6)	O9—O14—O3^iv^	89.88 (8)
O5—O7—O10	101.99 (7)	O12^xi^—O14—O6^viii^	75.69 (8)
N3—O7—O13	82.94 (13)	O11^i^—O14—O6^viii^	102.46 (10)
O8—O7—O13	59.99 (7)	O8—O14—O6^viii^	103.01 (9)
O9—O7—O13	104.46 (10)	O9—O14—O6^viii^	62.42 (7)
Tm—O7—O13	49.20 (5)	O3^iv^—O14—O6^viii^	145.65 (9)
O12^vii^—O7—O13	122.65 (9)	O12^xi^—O14—H9	114 (3)
O12—O7—O13	96.65 (7)	O11^i^—O14—H9	115 (3)
O5—O7—O13	55.37 (6)	O8—O14—H9	50 (3)
O10—O7—O13	89.60 (7)	O9—O14—H9	80 (3)
N3—O8—O7	32.77 (10)	O3^iv^—O14—H9	10 (3)
N3—O8—O9	27.84 (10)	O6^viii^—O14—H9	137 (3)
O7—O8—O9	60.61 (8)	O12^xi^—O14—H10	104 (5)
N3—O8—Tm	92.77 (11)	O11^i^—O14—H10	120 (5)
O7—O8—Tm	60.13 (6)	O8—O14—H10	67 (4)
O9—O8—Tm	120.49 (8)	O9—O14—H10	25 (4)
N3—O8—O10	93.66 (14)	O3^iv^—O14—H10	112 (5)
O7—O8—O10	76.28 (8)	O6^viii^—O14—H10	37 (4)
O9—O8—O10	107.87 (10)	H9—O14—H10	103 (5)
Tm—O8—O10	52.43 (5)		

Symmetry codes: (i) −*x*, −*y*+1, −*z*+1; (ii) −*x*+1, −*y*, −*z*+1; (iii) *x*+1, *y*, *z*; (iv) −*x*+1, −*y*+1, −*z*+1; (v) −*x*+1, −*y*+1, −*z*; (vi) *x*+1, *y*−1, *z*; (vii) −*x*, −*y*+1, −*z*; (viii) *x*−1, *y*+1, *z*; (ix) *x*−1, *y*, *z*; (x) *x*, *y*−1, *z*; (xi) *x*, *y*+1, *z*.

### Thulium nitrate (TONi-2_223K) Hydrogen-bond geometry (Å, º)

**Table:**

*D*—H···*A*	*D*—H	H···*A*	*D*···*A*	*D*—H···*A*
O10—H1···O1^i^	0.82 (4)	2.03 (4)	2.849 (2)	174 (3)
O10—H2···O13^ix^	0.78 (4)	2.32 (4)	2.996 (3)	145 (3)
O10—H2···O2^ix^	0.78 (4)	2.48 (4)	3.035 (3)	129 (3)
O11—H3···O14^i^	0.77 (5)	1.98 (5)	2.739 (3)	167 (5)
O11—H4···O2^ii^	0.85 (4)	2.03 (4)	2.874 (2)	171 (4)
O12—H5···O14^x^	0.83 (4)	1.90 (4)	2.715 (3)	165 (4)
O12—H6···O7^vii^	0.83 (4)	1.95 (4)	2.776 (2)	174 (4)
O13—H7···O5^v^	0.82 (5)	1.98 (5)	2.784 (2)	166 (4)
O13—H8···O3^iv^	0.86 (4)	2.12 (4)	2.953 (3)	163 (4)
O14—H9···O3^iv^	0.84 (4)	2.27 (5)	3.095 (3)	167 (4)
O14—H10···O9	0.68 (6)	2.44 (6)	3.040 (3)	148 (5)
O14—H10···O6^viii^	0.68 (6)	2.62 (6)	3.132 (4)	134 (5)

Symmetry codes: (i) −*x*, −*y*+1, −*z*+1; (ii) −*x*+1, −*y*, −*z*+1; (iv) −*x*+1, −*y*+1, −*z*+1; (v) −*x*+1, −*y*+1, −*z*; (vii) −*x*, −*y*+1, −*z*; (viii) *x*−1, *y*+1, *z*; (ix) *x*−1, *y*, *z*; (x) *x*, *y*−1, *z*.

### Thulium nitrate (TONii-3_223K) Crystal data

**Table:**

[Tm(NO_3_)_3_(H_2_O)_4_]·2H_2_O	*Z* = 2
*M**_r_* = 463.06	*F*(000) = 444
Triclinic, *P*1	*D*_x_ = 2.538 Mg m^−^^3^
*a* = 6.7050 (3) Å	Mo *K*α radiation, λ = 0.71073 Å
*b* = 8.9733 (4) Å	Cell parameters from 47483 reflections
*c* = 11.4915 (6) Å	θ = 2.6–36.6°
α = 70.924 (4)°	µ = 7.41 mm^−^^1^
β = 88.908 (4)°	*T* = 223 K
γ = 68.923 (4)°	Block, colourless
*V* = 605.90 (5) Å^3^	0.4 × 0.2 × 0.15 mm

### Thulium nitrate (TONii-3_223K) Data collection

**Table:**

STOE StadiVari diffractometer	4385 independent reflections
Radiation source: Genix 3D HF Mo	3899 reflections with *I* > 2σ(*I*)
Graded multilayer mirror monochromator	*R*_int_ = 0.031
Detector resolution: 5.81 pixels mm^-1^	θ_max_ = 32.5°, θ_min_ = 3.3°
ω scans	*h* = −10→10
Absorption correction: empirical (using intensity measurements) (X-AREA; Stoe, 2015)	*k* = −13→13
*T*_min_ = 0.615, *T*_max_ = 1.000	*l* = −17→17
29880 measured reflections	

### Thulium nitrate (TONii-3_223K) Refinement

**Table:**

Refinement on *F*^2^	Secondary atom site location: difference Fourier map
Least-squares matrix: full	Hydrogen site location: difference Fourier map
*R*[*F*^2^ > 2σ(*F*^2^)] = 0.016	All H-atom parameters refined
*wR*(*F*^2^) = 0.035	*w* = 1/[σ^2^(*F*_o_^2^) + (0.020*P*)^2^] where *P* = (*F*_o_^2^ + 2*F*_c_^2^)/3
*S* = 0.91	(Δ/σ)_max_ < 0.001
4385 reflections	Δρ_max_ = 1.11 e Å^−^^3^
221 parameters	Δρ_min_ = −1.18 e Å^−^^3^
0 restraints	Extinction correction: SHELXL-2014/7 (Sheldrick, 2015), Fc^*^=kFc[1+0.001xFc^2^λ^3^/sin(2θ)]^-1/4^
Primary atom site location: structure-invariant direct methods	Extinction coefficient: 0.0687 (8)

### Thulium nitrate (TONii-3_223K) Special details

**Table:**

Geometry. All esds (except the esd in the dihedral angle between two l.s. planes) are estimated using the full covariance matrix. The cell esds are taken into account individually in the estimation of esds in distances, angles and torsion angles; correlations between esds in cell parameters are only used when they are defined by crystal symmetry. An approximate (isotropic) treatment of cell esds is used for estimating esds involving l.s. planes.

### Thulium nitrate (TONii-3_223K) Fractional atomic coordinates and isotropic or equivalent isotropic displacement parameters (Å^2^)

**Table:**

	*x*	*y*	*z*	*U*_iso_*/*U*_eq_	
Tm	0.19543 (2)	0.41275 (2)	0.27232 (2)	0.01566 (4)	
N1	−0.1763 (3)	0.7245 (2)	0.18627 (18)	0.0219 (3)	
O1	−0.1088 (3)	0.6386 (2)	0.29939 (16)	0.0306 (4)	
O2	−0.0716 (3)	0.6650 (2)	0.10973 (17)	0.0332 (4)	
O3	−0.3348 (3)	0.8577 (2)	0.15483 (19)	0.0338 (4)	
N2	0.0790 (3)	0.1833 (2)	0.18432 (18)	0.0239 (4)	
O4	0.2565 (3)	0.1444 (2)	0.24492 (17)	0.0283 (3)	
O5	−0.0588 (3)	0.3320 (2)	0.16876 (17)	0.0282 (3)	
O6	0.0422 (4)	0.0843 (2)	0.14426 (19)	0.0358 (4)	
N3	0.0931 (3)	0.1599 (2)	0.50588 (18)	0.0216 (3)	
O7	−0.0255 (3)	0.2987 (2)	0.42350 (16)	0.0267 (3)	
O8	0.2889 (3)	0.1045 (3)	0.4993 (2)	0.0356 (4)	
O9	0.0120 (3)	0.0838 (2)	0.59033 (18)	0.0367 (4)	
O10	0.3559 (3)	0.6083 (2)	0.22157 (17)	0.0282 (4)	
H1	0.361 (6)	0.677 (5)	0.151 (4)	0.049 (10)*	
H2	0.385 (7)	0.637 (6)	0.269 (4)	0.051 (11)*	
O11	0.2745 (3)	0.4539 (2)	0.45290 (15)	0.0234 (3)	
H3	0.200 (7)	0.521 (5)	0.484 (4)	0.048 (11)*	
H4	0.367 (6)	0.390 (5)	0.499 (3)	0.034 (9)*	
O12	0.5603 (3)	0.2458 (2)	0.32343 (18)	0.0251 (3)	
H5	0.606 (6)	0.147 (5)	0.366 (3)	0.037 (9)*	
H6	0.652 (7)	0.283 (5)	0.310 (4)	0.049 (11)*	
O13	0.3038 (3)	0.4359 (2)	0.07487 (15)	0.0230 (3)	
H7	0.400 (7)	0.363 (5)	0.063 (4)	0.046 (10)*	
H8	0.236 (7)	0.500 (6)	0.006 (4)	0.059 (12)*	
O14	0.3663 (3)	0.8009 (2)	−0.01622 (17)	0.0272 (3)	
H9	0.252 (7)	0.832 (5)	−0.055 (4)	0.039 (10)*	
H10	0.391 (7)	0.883 (6)	−0.019 (4)	0.058 (12)*	
O15	0.4146 (3)	0.7754 (2)	0.36863 (17)	0.0244 (3)	
H11	0.301 (7)	0.813 (6)	0.390 (4)	0.052 (12)*	
H12	0.439 (8)	0.856 (6)	0.327 (4)	0.066 (14)*	

### Thulium nitrate (TONii-3_223K) Atomic displacement parameters (Å^2^)

**Table:**

	*U*^11^	*U*^22^	*U*^33^	*U*^12^	*U*^13^	*U*^23^
Tm	0.01512 (5)	0.01444 (4)	0.01475 (5)	−0.00368 (3)	0.00155 (2)	−0.00370 (3)
N1	0.0208 (8)	0.0134 (7)	0.0251 (9)	−0.0038 (6)	0.0013 (7)	−0.0012 (6)
O1	0.0303 (9)	0.0238 (8)	0.0203 (8)	0.0029 (7)	0.0054 (6)	−0.0003 (6)
O2	0.0341 (9)	0.0324 (9)	0.0227 (8)	0.0000 (7)	0.0010 (7)	−0.0103 (7)
O3	0.0260 (8)	0.0168 (7)	0.0389 (10)	0.0031 (6)	0.0023 (7)	0.0028 (7)
N2	0.0287 (9)	0.0200 (8)	0.0211 (8)	−0.0114 (7)	−0.0005 (7)	−0.0019 (7)
O4	0.0266 (8)	0.0189 (7)	0.0328 (9)	−0.0058 (6)	−0.0084 (7)	−0.0028 (6)
O5	0.0235 (8)	0.0266 (8)	0.0298 (8)	−0.0052 (6)	−0.0022 (6)	−0.0081 (7)
O6	0.0485 (12)	0.0275 (9)	0.0345 (10)	−0.0192 (8)	−0.0063 (8)	−0.0084 (7)
N3	0.0214 (8)	0.0162 (7)	0.0242 (9)	−0.0049 (6)	0.0059 (7)	−0.0056 (6)
O7	0.0282 (8)	0.0178 (7)	0.0252 (8)	−0.0058 (6)	0.0035 (6)	0.0004 (6)
O8	0.0191 (8)	0.0347 (10)	0.0532 (12)	−0.0060 (7)	0.0103 (8)	−0.0200 (9)
O9	0.0394 (10)	0.0256 (8)	0.0320 (9)	−0.0102 (8)	0.0145 (8)	0.0039 (7)
O10	0.0495 (11)	0.0276 (8)	0.0170 (7)	−0.0257 (8)	0.0066 (7)	−0.0073 (6)
O11	0.0259 (8)	0.0216 (7)	0.0160 (7)	−0.0025 (6)	−0.0007 (6)	−0.0050 (6)
O12	0.0169 (7)	0.0180 (7)	0.0331 (9)	−0.0032 (6)	−0.0001 (6)	−0.0029 (6)
O13	0.0257 (8)	0.0220 (7)	0.0165 (7)	−0.0040 (6)	0.0027 (6)	−0.0061 (6)
O14	0.0336 (9)	0.0208 (8)	0.0258 (8)	−0.0112 (7)	0.0027 (7)	−0.0049 (6)
O15	0.0267 (8)	0.0183 (7)	0.0249 (8)	−0.0065 (6)	−0.0009 (7)	−0.0050 (6)

### Thulium nitrate (TONii-3_223K) Geometric parameters (Å, º)

**Table:**

Tm—O10	2.2897 (18)	O7—O8	2.162 (3)
Tm—O11	2.3255 (17)	O7—O9	2.171 (2)
Tm—O12	2.3276 (17)	O7—O11	2.907 (3)
Tm—O13	2.3360 (16)	O7—O11^i^	3.006 (2)
Tm—O1	2.4039 (17)	O8—O9	2.151 (3)
Tm—O4	2.4179 (18)	O8—O8^vii^	2.774 (4)
Tm—O7	2.4677 (17)	O8—O12^vii^	2.943 (3)
Tm—O2	2.5034 (18)	O8—O11	2.969 (3)
Tm—O5	2.5252 (18)	O8—O12	2.974 (3)
Tm—N1	2.8787 (18)	O8—O15^viii^	3.184 (3)
Tm—N2	2.902 (2)	O9—O15^i^	2.782 (3)
Tm—O8	2.991 (2)	O9—O9^vi^	2.970 (5)
Tm—N3	3.1452 (18)	O9—O6^vi^	3.009 (3)
N1—O3	1.227 (2)	O10—O11	2.714 (2)
N1—O2	1.251 (3)	O10—O15	2.714 (3)
N1—O1	1.267 (2)	O10—O14	2.730 (3)
O1—O2	2.134 (2)	O10—O13	2.734 (2)
O1—O3	2.177 (2)	O10—O12	2.863 (3)
O1—O7	2.758 (2)	O10—H1	0.85 (4)
O1—O11	2.768 (3)	O10—H2	0.73 (5)
O1—O11^i^	3.004 (2)	O11—O15^viii^	2.666 (2)
O1—O10	3.166 (3)	O11—O12	2.917 (3)
O1—O15^ii^	3.174 (3)	O11—O1^i^	3.004 (2)
O2—O3	2.175 (3)	O11—O7^i^	3.006 (2)
O2—O13	2.738 (3)	O11—O15	3.199 (3)
O2—O5	2.809 (3)	O11—H3	0.81 (4)
O2—O10	2.965 (3)	O11—H4	0.74 (4)
O2—O6^iii^	3.102 (3)	O12—O8^vii^	2.943 (3)
O2—O13^iii^	3.184 (3)	O12—O13	2.986 (3)
O3—O14^iv^	2.885 (2)	O12—O9^vii^	3.170 (3)
O3—O15^ii^	2.992 (3)	O12—H5	0.80 (4)
O3—O14^ii^	3.111 (3)	O12—H6	0.79 (4)
N2—O6	1.221 (3)	O13—O14^ix^	2.713 (3)
N2—O4	1.263 (3)	O13—O5^iii^	2.963 (2)
N2—O5	1.277 (3)	O13—O2^iii^	3.184 (3)
O4—O5	2.148 (2)	O13—H7	0.78 (4)
O4—O6	2.173 (3)	O13—H8	0.83 (5)
O4—O12	2.781 (3)	O14—O13^ix^	2.713 (3)
O4—O13	2.827 (2)	O14—O6^iii^	2.804 (3)
O4—O8	2.829 (3)	O14—O3^iv^	2.885 (2)
O4—O15^v^	2.926 (2)	O14—O3^x^	3.111 (3)
O4—O7	3.087 (3)	O14—H9	0.80 (4)
O5—O6	2.190 (3)	O14—H10	0.80 (5)
O5—O7	2.849 (3)	O15—O11^viii^	2.666 (2)
O5—O13^iii^	2.963 (2)	O15—O9^i^	2.782 (3)
O5—O13	2.969 (2)	O15—O4^xi^	2.926 (2)
O6—O14^iii^	2.804 (3)	O15—O3^x^	2.992 (3)
O6—O9^vi^	3.009 (3)	O15—O1^x^	3.174 (3)
O6—O2^iii^	3.102 (3)	O15—O8^viii^	3.184 (3)
N3—O9	1.232 (2)	O15—H11	0.79 (5)
N3—O8	1.236 (3)	O15—H12	0.79 (5)
N3—O7	1.275 (2)		
			
O10—Tm—O11	72.03 (6)	O7—O8—O12	100.29 (9)
O10—Tm—O12	76.62 (7)	O8^vii^—O8—O12	61.49 (8)
O11—Tm—O12	77.63 (7)	O4—O8—O12	57.20 (6)
O10—Tm—O13	72.46 (6)	O12^vii^—O8—O12	124.10 (7)
O11—Tm—O13	141.28 (6)	O11—O8—O12	58.78 (6)
O12—Tm—O13	79.64 (7)	N3—O8—Tm	85.50 (14)
O10—Tm—O1	84.81 (7)	O9—O8—Tm	114.83 (9)
O11—Tm—O1	71.62 (6)	O7—O8—Tm	54.39 (6)
O12—Tm—O1	147.76 (7)	O8^vii^—O8—Tm	106.06 (10)
O13—Tm—O1	119.66 (6)	O4—O8—Tm	49.00 (5)
O10—Tm—O4	136.37 (7)	O12^vii^—O8—Tm	164.48 (8)
O11—Tm—O4	127.07 (6)	O11—O8—Tm	45.93 (4)
O12—Tm—O4	71.71 (6)	O12—O8—Tm	45.93 (5)
O13—Tm—O4	72.95 (6)	N3—O8—O15^viii^	118.44 (15)
O1—Tm—O4	136.08 (7)	O9—O8—O15^viii^	114.99 (11)
O10—Tm—O7	142.62 (6)	O7—O8—O15^viii^	113.47 (9)
O11—Tm—O7	74.59 (6)	O8^vii^—O8—O15^viii^	73.26 (9)
O12—Tm—O7	112.05 (6)	O4—O8—O15^viii^	125.89 (8)
O13—Tm—O7	143.65 (6)	O12^vii^—O8—O15^viii^	95.36 (8)
O1—Tm—O7	68.96 (6)	O11—O8—O15^viii^	51.20 (6)
O4—Tm—O7	78.37 (6)	O12—O8—O15^viii^	68.83 (7)
O10—Tm—O2	76.28 (7)	Tm—O8—O15^viii^	90.93 (6)
O11—Tm—O2	116.27 (6)	N3—O9—O8	29.45 (12)
O12—Tm—O2	143.26 (7)	N3—O9—O7	30.60 (11)
O13—Tm—O2	68.82 (6)	O8—O9—O7	60.05 (9)
O1—Tm—O2	51.50 (6)	N3—O9—O15^i^	122.54 (15)
O4—Tm—O2	114.16 (6)	O8—O9—O15^i^	151.29 (11)
O7—Tm—O2	104.56 (6)	O7—O9—O15^i^	92.26 (9)
O10—Tm—O5	138.32 (6)	N3—O9—O9^vi^	81.16 (15)
O11—Tm—O5	143.50 (6)	O8—O9—O9^vi^	81.92 (10)
O12—Tm—O5	122.22 (6)	O7—O9—O9^vi^	82.92 (10)
O13—Tm—O5	75.17 (6)	O15^i^—O9—O9^vi^	103.45 (11)
O1—Tm—O5	89.05 (6)	N3—O9—O6^vi^	155.54 (18)
O4—Tm—O5	51.46 (6)	O8—O9—O6^vi^	132.39 (12)
O7—Tm—O5	69.56 (6)	O7—O9—O6^vi^	154.89 (11)
O2—Tm—O5	67.92 (6)	O15^i^—O9—O6^vi^	70.34 (7)
O10—Tm—N1	79.34 (6)	O9^vi^—O9—O6^vi^	117.98 (10)
O11—Tm—N1	94.01 (6)	Tm—O10—O11	54.60 (5)
O12—Tm—N1	155.94 (6)	Tm—O10—O15	125.53 (8)
O13—Tm—N1	94.17 (6)	O11—O10—O15	72.21 (7)
O1—Tm—N1	25.80 (6)	Tm—O10—O14	123.63 (9)
O4—Tm—N1	128.96 (6)	O11—O10—O14	170.18 (10)
O7—Tm—N1	86.74 (5)	O15—O10—O14	106.61 (8)
O2—Tm—N1	25.70 (6)	Tm—O10—O13	54.55 (5)
O5—Tm—N1	77.55 (6)	O11—O10—O13	107.65 (8)
O10—Tm—N2	143.67 (6)	O15—O10—O13	179.02 (11)
O11—Tm—N2	142.17 (6)	O14—O10—O13	73.69 (7)
O12—Tm—N2	96.51 (6)	Tm—O10—O12	52.28 (5)
O13—Tm—N2	71.21 (6)	O11—O10—O12	63.00 (7)
O1—Tm—N2	113.66 (7)	O15—O10—O12	114.74 (9)
O4—Tm—N2	25.46 (6)	O14—O10—O12	124.80 (9)
O7—Tm—N2	73.26 (6)	O13—O10—O12	64.45 (7)
O2—Tm—N2	90.88 (6)	Tm—O10—O2	55.11 (6)
O5—Tm—N2	26.04 (6)	O11—O10—O2	92.39 (8)
N1—Tm—N2	103.50 (6)	O15—O10—O2	123.68 (9)
O10—Tm—O8	128.95 (6)	O14—O10—O2	80.12 (8)
O11—Tm—O8	66.54 (6)	O13—O10—O2	57.26 (6)
O12—Tm—O8	66.66 (6)	O12—O10—O2	103.77 (8)
O13—Tm—O8	129.82 (6)	Tm—O10—O1	49.12 (5)
O1—Tm—O8	108.14 (5)	O11—O10—O1	55.52 (6)
O4—Tm—O8	62.01 (6)	O15—O10—O1	93.03 (8)
O7—Tm—O8	45.43 (5)	O14—O10—O1	115.31 (9)
O2—Tm—O8	149.59 (6)	O13—O10—O1	87.66 (7)
O5—Tm—O8	92.10 (6)	O12—O10—O1	97.74 (7)
N1—Tm—O8	130.80 (5)	O2—O10—O1	40.57 (5)
N2—Tm—O8	76.75 (5)	Tm—O10—H1	130 (3)
O10—Tm—N3	140.57 (6)	O11—O10—H1	167 (3)
O11—Tm—N3	68.94 (6)	O15—O10—H1	100 (3)
O12—Tm—N3	89.70 (6)	O14—O10—H1	7 (3)
O13—Tm—N3	141.77 (6)	O13—O10—H1	81 (3)
O1—Tm—N3	88.19 (5)	O12—O10—H1	130 (3)
O4—Tm—N3	68.85 (6)	O2—O10—H1	84 (3)
O7—Tm—N3	22.36 (5)	O1—O10—H1	116 (3)
O2—Tm—N3	126.79 (6)	Tm—O10—H2	120 (3)
O5—Tm—N3	80.10 (6)	O11—O10—H2	65 (3)
N1—Tm—N3	108.51 (5)	O15—O10—H2	11 (3)
N2—Tm—N3	73.74 (5)	O14—O10—H2	115 (3)
O8—Tm—N3	23.07 (5)	O13—O10—H2	168 (3)
O3—N1—O2	122.6 (2)	O12—O10—H2	103 (3)
O3—N1—O1	121.5 (2)	O2—O10—H2	131 (3)
O2—N1—O1	115.82 (18)	O1—O10—H2	96 (3)
O3—N1—Tm	177.14 (17)	H1—O10—H2	108 (4)
O2—N1—Tm	60.15 (11)	Tm—O11—O15^viii^	123.70 (8)
O1—N1—Tm	55.68 (10)	Tm—O11—O10	53.37 (5)
N1—O1—O2	31.87 (11)	O15^viii^—O11—O10	122.60 (9)
N1—O1—O3	28.72 (11)	Tm—O11—O1	55.51 (5)
O2—O1—O3	60.58 (9)	O15^viii^—O11—O1	164.63 (9)
N1—O1—Tm	98.52 (12)	O10—O11—O1	70.56 (7)
O2—O1—Tm	66.66 (7)	Tm—O11—O7	54.93 (5)
O3—O1—Tm	127.24 (9)	O15^viii^—O11—O7	107.83 (8)
N1—O1—O7	128.53 (15)	O10—O11—O7	106.57 (8)
O2—O1—O7	106.29 (10)	O1—O11—O7	58.11 (6)
O3—O1—O7	140.60 (12)	Tm—O11—O12	51.22 (5)
Tm—O1—O7	56.61 (5)	O15^viii^—O11—O12	77.11 (7)
N1—O1—O11	139.90 (14)	O10—O11—O12	60.99 (6)
O2—O1—O11	112.99 (9)	O1—O11—O12	106.16 (7)
O3—O1—O11	155.01 (11)	O7—O11—O12	86.16 (7)
Tm—O1—O11	52.87 (5)	Tm—O11—O8	67.52 (6)
O7—O1—O11	63.47 (6)	O15^viii^—O11—O8	68.58 (7)
N1—O1—O11^i^	140.49 (14)	O10—O11—O8	114.24 (8)
O2—O1—O11^i^	158.78 (12)	O1—O11—O8	99.54 (8)
O3—O1—O11^i^	115.14 (9)	O7—O11—O8	43.17 (6)
Tm—O1—O11^i^	114.13 (7)	O12—O11—O8	60.70 (6)
O7—O1—O11^i^	62.71 (6)	Tm—O11—O1^i^	129.79 (8)
O11—O1—O11^i^	79.48 (7)	O15^viii^—O11—O1^i^	67.77 (7)
N1—O1—O10	86.14 (13)	O10—O11—O1^i^	167.02 (9)
O2—O1—O10	64.64 (8)	O1—O11—O1^i^	100.52 (7)
O3—O1—O10	106.16 (9)	O7—O11—O1^i^	74.86 (7)
Tm—O1—O10	46.07 (5)	O12—O11—O1^i^	131.78 (8)
O7—O1—O10	98.82 (7)	O8—O11—O1^i^	75.93 (7)
O11—O1—O10	53.92 (6)	Tm—O11—O7^i^	132.35 (8)
O11^i^—O1—O10	132.50 (8)	O15^viii^—O11—O7^i^	102.34 (7)
N1—O1—O15^ii^	89.65 (13)	O10—O11—O7^i^	113.06 (8)
O2—O1—O15^ii^	117.05 (9)	O1—O11—O7^i^	76.86 (7)
O3—O1—O15^ii^	64.92 (7)	O7—O11—O7^i^	102.68 (7)
Tm—O1—O15^ii^	150.29 (9)	O12—O11—O7^i^	170.75 (9)
O7—O1—O15^ii^	96.18 (7)	O8—O11—O7^i^	127.94 (8)
O11—O1—O15^ii^	129.57 (8)	O1^i^—O11—O7^i^	54.64 (5)
O11^i^—O1—O15^ii^	51.03 (5)	Tm—O11—O15	106.37 (7)
O10—O1—O15^ii^	163.64 (8)	O15^viii^—O11—O15	102.98 (7)
N1—O2—O1	32.31 (11)	O10—O11—O15	53.90 (6)
N1—O2—O3	28.38 (11)	O1—O11—O15	91.32 (7)
O1—O2—O3	60.69 (8)	O7—O11—O15	149.18 (8)
N1—O2—Tm	94.15 (13)	O12—O11—O15	100.27 (7)
O1—O2—Tm	61.84 (6)	O8—O11—O15	160.04 (8)
O3—O2—Tm	122.53 (9)	O1^i^—O11—O15	118.68 (7)
N1—O2—O13	145.64 (15)	O7^i^—O11—O15	70.75 (6)
O1—O2—O13	113.94 (9)	Tm—O11—H3	129 (3)
O3—O2—O13	170.33 (12)	O15^viii^—O11—H3	105 (3)
Tm—O2—O13	52.70 (5)	O10—O11—H3	113 (3)
N1—O2—O5	107.14 (15)	O1—O11—H3	74 (3)
O1—O2—O5	87.66 (9)	O7—O11—H3	99 (3)
O3—O2—O5	121.17 (11)	O12—O11—H3	173 (3)
Tm—O2—O5	56.41 (5)	O8—O11—H3	126 (3)
O13—O2—O5	64.70 (7)	O1^i^—O11—H3	54 (3)
N1—O2—O10	95.68 (14)	O7^i^—O11—H3	4 (3)
O1—O2—O10	74.80 (8)	O15—O11—H3	73 (3)
O3—O2—O10	113.21 (10)	Tm—O11—H4	121 (3)
Tm—O2—O10	48.61 (5)	O15^viii^—O11—H4	4 (3)
O13—O2—O10	57.13 (6)	O10—O11—H4	118 (3)
O5—O2—O10	102.39 (8)	O1—O11—H4	168 (3)
N1—O2—O6^iii^	118.54 (14)	O7—O11—H4	110 (3)
O1—O2—O6^iii^	143.35 (11)	O12—O11—H4	74 (3)
O3—O2—O6^iii^	93.77 (9)	O8—O11—H4	69 (3)
Tm—O2—O6^iii^	134.73 (9)	O1^i^—O11—H4	72 (3)
O13—O2—O6^iii^	86.94 (7)	O7^i^—O11—H4	105 (3)
O5—O2—O6^iii^	128.98 (8)	O15—O11—H4	101 (3)
O10—O2—O6^iii^	94.82 (8)	H3—O11—H4	108 (4)
N1—O2—O13^iii^	119.36 (15)	Tm—O12—O4	55.65 (5)
O1—O2—O13^iii^	129.73 (11)	Tm—O12—O10	51.09 (5)
O3—O2—O13^iii^	103.41 (9)	O4—O12—O10	101.51 (7)
Tm—O2—O13^iii^	113.12 (7)	Tm—O12—O11	51.15 (5)
O13—O2—O13^iii^	86.20 (7)	O4—O12—O11	96.35 (7)
O5—O2—O13^iii^	58.87 (6)	O10—O12—O11	56.01 (6)
O10—O2—O13^iii^	143.20 (8)	Tm—O12—O8^vii^	121.45 (8)
O6^iii^—O2—O13^iii^	78.86 (6)	O4—O12—O8^vii^	81.85 (7)
N1—O3—O2	28.99 (12)	O10—O12—O8^vii^	161.11 (9)
N1—O3—O1	29.74 (11)	O11—O12—O8^vii^	105.28 (8)
O2—O3—O1	58.73 (8)	Tm—O12—O8	67.41 (6)
N1—O3—O14^iv^	129.45 (15)	O4—O12—O8	58.77 (7)
O2—O3—O14^iv^	112.88 (10)	O10—O12—O8	109.76 (8)
O1—O3—O14^iv^	136.24 (11)	O11—O12—O8	60.52 (6)
N1—O3—O15^ii^	99.15 (14)	O8^vii^—O12—O8	55.90 (7)
O2—O3—O15^ii^	123.07 (9)	Tm—O12—O13	50.30 (5)
O1—O3—O15^ii^	73.86 (8)	O4—O12—O13	58.57 (6)
O14^iv^—O3—O15^ii^	123.21 (8)	O10—O12—O13	55.69 (6)
N1—O3—O14^ii^	103.78 (16)	O11—O12—O13	96.31 (7)
O2—O3—O14^ii^	86.31 (9)	O8^vii^—O12—O13	136.79 (9)
O1—O3—O14^ii^	119.12 (10)	O8—O12—O13	108.33 (8)
O14^iv^—O3—O14^ii^	101.62 (7)	Tm—O12—O9^vii^	159.42 (9)
O15^ii^—O3—O14^ii^	91.31 (7)	O4—O12—O9^vii^	105.31 (8)
O6—N2—O4	122.0 (2)	O10—O12—O9^vii^	149.31 (9)
O6—N2—O5	122.5 (2)	O11—O12—O9^vii^	133.55 (8)
O4—N2—O5	115.49 (19)	O8^vii^—O12—O9^vii^	40.99 (5)
O6—N2—Tm	175.95 (18)	O8—O12—O9^vii^	96.86 (7)
O4—N2—Tm	55.34 (11)	O13—O12—O9^vii^	130.07 (8)
O5—N2—Tm	60.28 (11)	Tm—O12—H5	124 (3)
N2—O4—O5	32.44 (11)	O4—O12—H5	80 (3)
N2—O4—O6	28.47 (12)	O10—O12—H5	167 (3)
O5—O4—O6	60.91 (9)	O11—O12—H5	111 (3)
N2—O4—Tm	99.20 (13)	O8^vii^—O12—H5	6 (3)
O5—O4—Tm	66.85 (7)	O8—O12—H5	60 (3)
O6—O4—Tm	127.60 (9)	O13—O12—H5	133 (3)
N2—O4—O12	149.28 (14)	O9^vii^—O12—H5	38 (3)
O5—O4—O12	118.57 (9)	Tm—O12—H6	124 (3)
O6—O4—O12	167.51 (11)	O4—O12—H6	151 (3)
Tm—O4—O12	52.63 (5)	O10—O12—H6	73 (3)
N2—O4—O13	89.39 (12)	O11—O12—H6	103 (3)
O5—O4—O13	71.81 (8)	O8^vii^—O12—H6	113 (3)
O6—O4—O13	105.26 (9)	O8—O12—H6	150 (3)
Tm—O4—O13	52.19 (5)	O13—O12—H6	98 (3)
O12—O4—O13	64.35 (6)	O9^vii^—O12—H6	76 (3)
N2—O4—O8	121.99 (15)	H5—O12—H6	112 (4)
O5—O4—O8	105.54 (10)	Tm—O13—O14^ix^	126.06 (8)
O6—O4—O8	128.45 (10)	Tm—O13—O10	52.99 (5)
Tm—O4—O8	68.99 (6)	O14^ix^—O13—O10	123.85 (9)
O12—O4—O8	64.03 (7)	Tm—O13—O2	58.48 (5)
O13—O4—O8	117.39 (8)	O14^ix^—O13—O2	170.54 (10)
N2—O4—O15^v^	105.48 (13)	O10—O13—O2	65.61 (7)
O5—O4—O15^v^	132.47 (10)	Tm—O13—O4	54.86 (5)
O6—O4—O15^v^	80.38 (8)	O14^ix^—O13—O4	82.46 (7)
Tm—O4—O15^v^	144.77 (8)	O10—O13—O4	103.61 (7)
O12—O4—O15^v^	105.17 (7)	O2—O13—O4	95.85 (8)
O13—O4—O15^v^	150.26 (9)	Tm—O13—O5^iii^	128.52 (8)
O8—O4—O15^v^	76.65 (7)	O14^ix^—O13—O5^iii^	103.47 (7)
N2—O4—O7	84.55 (14)	O10—O13—O5^iii^	112.21 (8)
O5—O4—O7	62.95 (8)	O2—O13—O5^iii^	70.49 (7)
O6—O4—O7	104.27 (9)	O4—O13—O5^iii^	130.25 (8)
Tm—O4—O7	51.53 (5)	Tm—O13—O5	55.31 (5)
O12—O4—O7	85.18 (7)	O14^ix^—O13—O5	115.55 (8)
O13—O4—O7	100.94 (7)	O10—O13—O5	104.14 (7)
O8—O4—O7	42.60 (6)	O2—O13—O5	58.80 (7)
O15^v^—O4—O7	105.93 (7)	O4—O13—O5	43.43 (6)
N2—O5—O4	32.07 (11)	O5^iii^—O13—O5	93.63 (7)
N2—O5—O6	28.04 (11)	Tm—O13—O12	50.06 (5)
O4—O5—O6	60.10 (8)	O14^ix^—O13—O12	80.61 (7)
N2—O5—Tm	93.68 (13)	O10—O13—O12	59.86 (6)
O4—O5—Tm	61.69 (7)	O2—O13—O12	106.36 (7)
O6—O5—Tm	121.66 (9)	O4—O13—O12	57.07 (6)
N2—O5—O2	138.74 (15)	O5^iii^—O13—O12	171.66 (8)
O4—O5—O2	112.22 (9)	O5—O13—O12	91.06 (7)
O6—O5—O2	153.44 (11)	Tm—O13—O2^iii^	119.90 (8)
Tm—O5—O2	55.67 (5)	O14^ix^—O13—O2^iii^	76.74 (7)
N2—O5—O7	95.06 (13)	O10—O13—O2^iii^	159.18 (9)
O4—O5—O7	74.85 (8)	O2—O13—O2^iii^	93.80 (7)
O6—O5—O7	111.87 (9)	O4—O13—O2^iii^	80.58 (7)
Tm—O5—O7	54.27 (5)	O5^iii^—O13—O2^iii^	54.24 (6)
O2—O5—O7	88.06 (7)	O5—O13—O2^iii^	64.66 (6)
N2—O5—O13^iii^	122.46 (14)	O12—O13—O2^iii^	134.09 (8)
O4—O5—O13^iii^	139.71 (10)	Tm—O13—H7	122 (3)
O6—O5—O13^iii^	101.74 (9)	O14^ix^—O13—H7	5 (3)
Tm—O5—O13^iii^	120.11 (8)	O10—O13—H7	122 (3)
O2—O5—O13^iii^	66.89 (6)	O2—O13—H7	171 (3)
O7—O5—O13^iii^	142.29 (8)	O4—O13—H7	78 (3)
N2—O5—O13	82.93 (13)	O5^iii^—O13—H7	108 (3)
O4—O5—O13	64.76 (7)	O5—O13—H7	113 (3)
O6—O5—O13	100.34 (9)	O12—O13—H7	77 (3)
Tm—O5—O13	49.52 (5)	O2^iii^—O13—H7	78 (3)
O2—O5—O13	56.50 (6)	Tm—O13—H8	129 (3)
O7—O5—O13	103.35 (7)	O14^ix^—O13—H8	103 (3)
O13^iii^—O5—O13	86.37 (7)	O10—O13—H8	113 (3)
N2—O6—O4	29.55 (12)	O2—O13—H8	71 (3)
N2—O6—O5	29.44 (12)	O4—O13—H8	130 (3)
O4—O6—O5	58.99 (8)	O5^iii^—O13—H8	1 (3)
N2—O6—O14^iii^	116.45 (16)	O5—O13—H8	94 (3)
O4—O6—O14^iii^	146.00 (11)	O12—O13—H8	172 (3)
O5—O6—O14^iii^	87.01 (9)	O2^iii^—O13—H8	54 (3)
N2—O6—O9^vi^	83.84 (14)	H7—O13—H8	107 (4)
O4—O6—O9^vi^	77.76 (9)	O13^ix^—O14—O10	98.12 (8)
O5—O6—O9^vi^	91.17 (9)	O13^ix^—O14—O6^iii^	118.03 (9)
O14^iii^—O6—O9^vi^	104.35 (9)	O10—O14—O6^iii^	107.66 (9)
N2—O6—O2^iii^	83.25 (14)	O13^ix^—O14—O3^iv^	112.05 (8)
O4—O6—O2^iii^	93.56 (9)	O10—O14—O3^iv^	139.09 (9)
O5—O6—O2^iii^	74.80 (8)	O6^iii^—O14—O3^iv^	82.46 (7)
O14^iii^—O6—O2^iii^	76.63 (7)	O13^ix^—O14—O3^x^	93.53 (8)
O9^vi^—O6—O2^iii^	165.92 (9)	O10—O14—O3^x^	72.61 (7)
O9—N3—O8	121.2 (2)	O6^iii^—O14—O3^x^	147.52 (9)
O9—N3—O7	119.94 (19)	O3^iv^—O14—O3^x^	78.38 (7)
O8—N3—O7	118.84 (19)	O13^ix^—O14—O13	69.68 (7)
O9—N3—Tm	167.34 (15)	O10—O14—O13	53.22 (6)
O8—N3—Tm	71.43 (13)	O6^iii^—O14—O13	82.55 (7)
O7—N3—Tm	47.41 (10)	O3^iv^—O14—O13	163.54 (9)
N3—O7—O8	30.06 (11)	O3^x^—O14—O13	118.06 (7)
N3—O7—O9	29.46 (11)	O13^ix^—O14—H9	116 (3)
O8—O7—O9	59.52 (9)	O10—O14—H9	109 (3)
N3—O7—Tm	110.23 (13)	O6^iii^—O14—H9	3 (3)
O8—O7—Tm	80.17 (8)	O3^iv^—O14—H9	83 (3)
O9—O7—Tm	139.69 (10)	O3^x^—O14—H9	150 (3)
N3—O7—O1	147.87 (15)	O13—O14—H9	82 (3)
O8—O7—O1	125.43 (10)	O13^ix^—O14—H10	114 (3)
O9—O7—O1	152.51 (11)	O10—O14—H10	111 (3)
Tm—O7—O1	54.43 (5)	O6^iii^—O14—H10	108 (3)
N3—O7—O5	121.52 (14)	O3^iv^—O14—H10	31 (3)
O8—O7—O5	104.49 (9)	O3^x^—O14—H10	48 (3)
O9—O7—O5	130.94 (10)	O13—O14—H10	164 (3)
Tm—O7—O5	56.17 (5)	H9—O14—H10	109 (4)
O1—O7—O5	76.12 (7)	O11^viii^—O15—O10	105.91 (8)
N3—O7—O11	89.75 (13)	O11^viii^—O15—O9^i^	123.52 (9)
O8—O7—O11	69.95 (8)	O10—O15—O9^i^	97.93 (9)
O9—O7—O11	109.13 (10)	O11^viii^—O15—O4^xi^	135.84 (8)
Tm—O7—O11	50.47 (5)	O10—O15—O4^xi^	113.20 (8)
O1—O7—O11	58.42 (6)	O9^i^—O15—O4^xi^	71.01 (7)
O5—O7—O11	106.35 (7)	O11^viii^—O15—O3^x^	101.79 (8)
N3—O7—O11^i^	109.28 (13)	O10—O15—O3^x^	74.76 (7)
O8—O7—O11^i^	123.31 (10)	O9^i^—O15—O3^x^	133.98 (8)
O9—O7—O11^i^	91.56 (9)	O4^xi^—O15—O3^x^	70.82 (7)
Tm—O7—O11^i^	112.11 (7)	O11^viii^—O15—O1^x^	61.19 (6)
O1—O7—O11^i^	62.65 (6)	O10—O15—O1^x^	80.98 (7)
O5—O7—O11^i^	128.92 (7)	O9^i^—O15—O1^x^	175.19 (8)
O11—O7—O11^i^	77.32 (7)	O4^xi^—O15—O1^x^	105.04 (8)
N3—O7—O4	83.39 (12)	O3^x^—O15—O1^x^	41.22 (5)
O8—O7—O4	62.31 (8)	O11^viii^—O15—O8^viii^	60.23 (6)
O9—O7—O4	105.04 (9)	O10—O15—O8^viii^	151.59 (9)
Tm—O7—O4	50.10 (4)	O9^i^—O15—O8^viii^	110.39 (8)
O1—O7—O4	99.67 (7)	O4^xi^—O15—O8^viii^	75.62 (6)
O5—O7—O4	42.20 (5)	O3^x^—O15—O8^viii^	83.73 (7)
O11—O7—O4	90.17 (7)	O1^x^—O15—O8^viii^	70.61 (6)
O11^i^—O7—O4	161.88 (7)	O11^viii^—O15—O11	77.02 (7)
N3—O8—O9	29.33 (11)	O10—O15—O11	53.89 (6)
N3—O8—O7	31.10 (11)	O9^i^—O15—O11	77.44 (7)
O9—O8—O7	60.43 (8)	O4^xi^—O15—O11	143.83 (8)
N3—O8—O8^vii^	164.0 (2)	O3^x^—O15—O11	124.74 (8)
O9—O8—O8^vii^	137.71 (14)	O1^x^—O15—O11	105.40 (7)
O7—O8—O8^vii^	157.96 (15)	O8^viii^—O15—O11	133.51 (8)
N3—O8—O4	95.90 (15)	O11^viii^—O15—H11	116 (3)
O9—O8—O4	114.74 (11)	O10—O15—H11	105 (3)
O7—O8—O4	75.09 (9)	O9^i^—O15—H11	8 (3)
O8^vii^—O8—O4	84.07 (10)	O4^xi^—O15—H11	73 (3)
N3—O8—O12^vii^	103.77 (15)	O3^x^—O15—H11	140 (3)
O9—O8—O12^vii^	75.16 (9)	O1^x^—O15—H11	174 (3)
O7—O8—O12^vii^	133.87 (10)	O8^viii^—O15—H11	104 (3)
O8^vii^—O8—O12^vii^	62.61 (9)	O11—O15—H11	78 (3)
O4—O8—O12^vii^	116.75 (8)	O11^viii^—O15—H12	116 (3)
N3—O8—O11	87.65 (14)	O10—O15—H12	108 (3)
O9—O8—O11	107.57 (9)	O9^i^—O15—H12	103 (3)
O7—O8—O11	66.87 (8)	O4^xi^—O15—H12	32 (3)
O8^vii^—O8—O11	108.35 (11)	O3^x^—O15—H12	43 (3)
O4—O8—O11	94.14 (8)	O1^x^—O15—H12	73 (3)
O12^vii^—O8—O11	145.13 (10)	O8^viii^—O15—H12	64 (3)
N3—O8—O12	131.37 (16)	O11—O15—H12	162 (3)
O9—O8—O12	160.61 (11)	H11—O15—H12	105 (5)

Symmetry codes: (i) −*x*, −*y*+1, −*z*+1; (ii) *x*−1, *y*, *z*; (iii) −*x*, −*y*+1, −*z*; (iv) −*x*, −*y*+2, −*z*; (v) *x*, *y*−1, *z*; (vi) −*x*, −*y*, −*z*+1; (vii) −*x*+1, −*y*, −*z*+1; (viii) −*x*+1, −*y*+1, −*z*+1; (ix) −*x*+1, −*y*+1, −*z*; (x) *x*+1, *y*, *z*; (xi) *x*, *y*+1, *z*.

### Thulium nitrate (TONii-3_223K) Hydrogen-bond geometry (Å, º)

**Table:**

*D*—H···*A*	*D*—H	H···*A*	*D*···*A*	*D*—H···*A*
O10—H1···O14	0.85 (4)	1.89 (4)	2.730 (3)	170 (4)
O10—H2···O15	0.73 (5)	2.00 (5)	2.714 (3)	164 (5)
O11—H4···O15^viii^	0.74 (4)	1.93 (4)	2.666 (2)	174 (4)
O11—H3···O7^i^	0.81 (4)	2.20 (4)	3.006 (2)	175 (4)
O12—H5···O8^vii^	0.80 (4)	2.15 (4)	2.943 (3)	172 (4)
O12—H6···O5^x^	0.79 (4)	2.57 (4)	3.260 (3)	147 (4)
O12—H6···O7^x^	0.79 (4)	2.61 (4)	3.269 (3)	142 (4)
O13—H7···O14^ix^	0.78 (4)	1.93 (4)	2.713 (3)	174 (4)
O13—H8···O5^iii^	0.83 (5)	2.13 (5)	2.963 (2)	179 (5)
O14—H9···O6^iii^	0.80 (4)	2.01 (4)	2.804 (3)	176 (4)
O14—H10···O3^x^	0.80 (5)	2.64 (5)	3.111 (3)	119 (4)
O14—H10···O3^iv^	0.80 (5)	2.24 (5)	2.885 (2)	138 (4)
O15—H11···O9^i^	0.79 (5)	2.01 (5)	2.782 (3)	168 (4)
O15—H12···O4^xi^	0.79 (5)	2.29 (5)	2.926 (2)	137 (4)
O15—H12···O3^x^	0.79 (5)	2.47 (5)	2.992 (3)	125 (4)

Symmetry codes: (i) −*x*, −*y*+1, −*z*+1; (iii) −*x*, −*y*+1, −*z*; (iv) −*x*, −*y*+2, −*z*; (vii) −*x*+1, −*y*, −*z*+1; (viii) −*x*+1, −*y*+1, −*z*+1; (ix) −*x*+1, −*y*+1, −*z*; (x) *x*+1, *y*, *z*; (xi) *x*, *y*+1, *z*.
